# Large language model processing capabilities of ChatGPT 4.0 to generate molecular tumor board recommendations—a critical evaluation on real world data

**DOI:** 10.1093/oncolo/oyaf293

**Published:** 2025-09-18

**Authors:** Maximilian Schmutz, Sebastian Sommer, Julia Sander, David Graumann, Johannes Raffler, Iñaki Soto-Rey, Seyedmostafa Sheikhalishahi, Lisa Schmidt, Leonhard Paul Unkelbach, Levent Ortak, Tina Schaller, Sebastian Dintner, Kathrin Hildebrand, Michaela Kuhlen, Frank Jordan, Martin Trepel, Christian Hinske, Rainer Claus

**Affiliations:** Department of Hematology and Oncology, Faculty of Medicine, University of Augsburg, Augsburg 86156, Germany; Department of Data Management and Clinical Decision Support, Faculty of Medicine, University of Augsburg, Augsburg 86356, Germany; Bavarian Cancer Research Center (BZKF), Erlangen 91054, Germany; Department of Hematology and Oncology, Faculty of Medicine, University of Augsburg, Augsburg 86156, Germany; Department of Data Management and Clinical Decision Support, Faculty of Medicine, University of Augsburg, Augsburg 86356, Germany; Department of Hematology and Oncology, Faculty of Medicine, University of Augsburg, Augsburg 86156, Germany; Department of Data Management and Clinical Decision Support, Faculty of Medicine, University of Augsburg, Augsburg 86356, Germany; Bavarian Cancer Research Center (BZKF), Erlangen 91054, Germany; Department of Data Management and Clinical Decision Support, Faculty of Medicine, University of Augsburg, Augsburg 86356, Germany; Bavarian Cancer Research Center (BZKF), Erlangen 91054, Germany; Department of Data Management and Clinical Decision Support, Faculty of Medicine, University of Augsburg, Augsburg 86356, Germany; Faculty of Medicine, University of Augsburg, Augsburg 86195, Germany; Faculty of Medicine, University of Augsburg, Augsburg 86195, Germany; Faculty of Medicine, University of Augsburg, Augsburg 86195, Germany; General Pathology and Molecular Diagnostics, Faculty of Medicine, University of Augsburg, Augsburg 86156, Germany; General Pathology and Molecular Diagnostics, Faculty of Medicine, University of Augsburg, Augsburg 86156, Germany; General Pathology and Molecular Diagnostics, Faculty of Medicine, University of Augsburg, Augsburg 86156, Germany; Pediatrics and Adolescent Medicine, Faculty of Medicine, University of Augsburg, Augsburg 86156, Germany; Department of Hematology and Oncology, Faculty of Medicine, University of Augsburg, Augsburg 86156, Germany; Department of Hematology and Oncology, Faculty of Medicine, University of Augsburg, Augsburg 86156, Germany; Bavarian Cancer Research Center (BZKF), Erlangen 91054, Germany; Comprehensive Cancer Center Augsburg (CCCA), Faculty of Medicine, University of Augsburg, Augsburg 86156, Germany; Department of Data Management and Clinical Decision Support, Faculty of Medicine, University of Augsburg, Augsburg 86356, Germany; Department of Hematology and Oncology, Faculty of Medicine, University of Augsburg, Augsburg 86156, Germany; Bavarian Cancer Research Center (BZKF), Erlangen 91054, Germany; General Pathology and Molecular Diagnostics, Faculty of Medicine, University of Augsburg, Augsburg 86156, Germany; Comprehensive Cancer Center Augsburg (CCCA), Faculty of Medicine, University of Augsburg, Augsburg 86156, Germany

**Keywords:** precision oncology, molecular tumor board, variant annotation, artificial intelligence, ChatGPT 4.0, large language models

## Abstract

**Background:**

Large language models (LLMs) like ChatGPT 4.0 hold promise for enhancing clinical decision-making in precision oncology, particularly within molecular tumor boards (MTBs). This study assesses ChatGPT 4.0’s performance in generating therapy recommendations for complex real-world cancer cases compared to expert human MTB (hMTB) teams.

**Methods:**

We retrospectively analyzed 20 anonymized MTB cases from the Comprehensive Cancer Center Augsburg (CCCA), covering breast cancer (*n* = 3), glioblastoma (*n* = 3), colorectal cancer (*n* = 2), and rare tumors. ChatGPT 4.0 recommendations were evaluated against hMTB outputs using metrics including recommendation type (therapeutic/diagnostic), information density (IDM), consistency, quality (level of evidence [LoE]), and efficiency. Each case was prompted thrice to evaluate variability (Fleiss’ Kappa).

**Results:**

ChatGPT 4.0 generated more therapeutic recommendations per case than hMTB (median 3 vs 1, *P* = .005), with comparable diagnostic suggestions (median 1 vs 2, *P* = .501). Therapeutic scope from ChatGPT 4.0 included off-label and clinical trial options. IDM scores indicated similar content depth between ChatGPT 4.0 (median 0.67) and hMTB (median 0.75; *P* = .084). Moderate consistency was observed across replicate runs (median Fleiss’ Kappa = 0.51). ChatGPT 4.0 occasionally utilized lower-level or preclinical evidence more frequently (*P* = .0019). Efficiency favored ChatGPT 4.0 significantly (median 15.2 vs 34.7 minutes; *P* < .001).

**Conclusion:**

Incorporating ChatGPT 4.0 into MTB workflows enhances efficiency and provides relevant recommendations, especially in guideline-supported cases. However, variability in evidence prioritization highlights the need for ongoing human oversight. A hybrid approach, integrating human expertise with LLM support, may optimize precision oncology decision-making.

Implications for Practice: The integration of large language models (LLMs) such as ChatGPT 4.0 into Molecular Tumor Board (MTB) workflows presents a significant opportunity to enhance efficiency and expand therapeutic considerations in precision oncology. While the model reliably generates guideline-concordant recommendations in standard cases and proposes additional therapeutic options in complex scenarios, its reliance on lower-level evidence in some outputs underscores the necessity for human oversight. A hybrid model—leveraging the speed and breadth of LLMs alongside expert clinical judgement—may improve decision-making processes, particularly in resource-constrained settings. However, clear validation frameworks, transparent citation of evidence, and institutional governance are essential to ensure safe and effective implementation.

## Introduction

Precision oncology tailors cancer treatments to individual tumor profiles by identifying specific molecular alterations that guide targeted therapy.^1–3^ However, the growing array of diagnostic tools—such as comprehensive genomic profiling and exome or genome sequencing—makes clinical annotation and evidence interpretation increasingly complex.^4^,^5^ Beyond molecular findings, patient context and preferences also influence therapeutic decisions.^3^ To address this complexity, Molecular Tumor Boards (MTBs) integrate multidisciplinary expertise, including oncology, pathology, genetics, and bioinformatics.^6^ Yet, the lack of universal standards for evaluating molecular data and recommending treatments results in significant variability among MTBs,^7^,^8^ driven partly by disparate data availability and interpretations.^9^ Multiple systems propose levels of evidence (LoE) for genetic aberrations, but consensus remains elusive.^10^^,^^11^

Recent advances in large language models (LLMs) like ChatGPT have sparked interest in their potential to streamline MTB workflows.^12^ These models can generate detailed, contextually relevant text and respond to complex queries, surpassing traditional search engines in certain scenarios.^13^,^14^ In principle, LLMs could assist MTBs by analyzing molecular data, identifying actionable mutations, and furnishing high-level summaries. Early studies suggest that AI chatbots sometimes match or exceed physicians in the quality and empathy of medical responses.^15^ Nonetheless, human expertise is indispensable for handling multidimensional clinical data and nuanced decision-making, as shown by recent research indicating that expert oversight remains critical in complex cases.^16^ Integrating LLMs into MTBs, thus, poses both opportunities for enhanced efficiency and risks if inaccuracies or misinterpretations occur, underscoring the need for careful assessment of model outputs.

This study explores how ChatGPT and similar LLMs might aid MTBs in annotating and interpreting post-last-line therapy biomarkers. We evaluate the reliability, quality, and efficiency of LLM-derived recommendations and discuss how these models could support—and not replace—expert judgment. As the demand for personalized care expands amid constrained ­clinical resources, leveraging LLMs may help optimize decision-making and advance precision oncology by improving access to comprehensive, evidence-based insights.

## Patients and methods

### Molecular tumor board at the Comprehensive Cancer Center Augsburg

The Molecular Tumor Board (MTB) at the Comprehensive Cancer Center Augsburg (CCCA) was established in 2018 to improve treatment outcomes for patients with advanced or rare malignancies lacking standard therapeutic options. The MTB convenes weekly, comprising interdisciplinary experts in oncology, pathology, molecular pathology, human genetics, and bioinformatics. After each discussion, the MTB issues structured therapeutic or diagnostic recommendations, together with an evidence summary, following the NCT-based levels of evidence (LoE) classification (1A–4A).^11^ Details are given in Table S1. Recommendations can assist in reimbursement applications and inform patient-specific treatment strategies.

### Patient selection and data

For this study, real, sequential MTB cases from June to August 2021 were included to match the ChatGPT 4.0 knowledge cutoff date in September 2021. Among these, 20 consecutive cases displayed at least one confirmed genetic aberration. All patients provided consent for anonymized, retrospective use of their data. No further ethics approval was required per Bavarian Hospital Act (BayKrG Art. 27 Para. 4). Patient demographics (eg, age, comorbidities) were not included, as therapy decisions primarily depended on tumor biology and prior treatments, and final therapeutic recommendations were at the discretion of the treating physician. Molecular profiling used multigene NGS panels (eg, AmpliSeq for Illumina Cancer HotSpot Panel v2), identifying SNVs, CNVs, or fusions. Immunohistochemistry was conducted if relevant (eg, for antibody-drug conjugates).

### Prompt engineering strategy

Clinical patient summaries and pathology reports were used to manually extract relevant data for hMTB case discussions. The same data was provided to the LLM via inclusion into a standardized prompt in the English language (Table S2). Extracted data are condensed into a summarized report form, on which the hMTB case discussion and preparation are based on. No other sources were integrated for the actual case discussion.

ChatGPT was informed that all outputs were for educational case discussions, not direct clinical use, prior to running the prompts. Each case was prompted in triplicates, and answers were copied to capture the variability between answers. It was noted to have misinterpretation or omission of critical information triggers a re-prompt with identical content by highlighting the omission or misinterpretation to the system, if necessary. An exemplary prompt is shown for case 18, which showed the highest Kappa Fleiss value as internally most consistent triplicate, alongside the first iteration of the corresponding answer. The complete transcript can be found in Table S3 alongside all other triplicate transcripts.

Prompts for querying a ChatGPT 4.0 were systematically structured to simulate multidisciplinary molecular tumor board (MTB) discussions. Each prompt positioned the LLM as a hematologist/oncologist collaborating with specialists in pathology, molecular pathology, human genetics, and pediatric oncology. Technical terminology was used. Detailed clinical context was provided, including disease diagnosis, UICC stage, histopathological features, comprehensive immunohistochemical (IHC) profiles (eg, TPS, CPS, ICS, ER, PR, HER2/neu, Ki-67, HPV status), microsatellite stability status (MSS/MSI), and specific molecular genetic alterations identified through targeted sequencing (AmpliSeq Focus Panel, AmpliSeq BRCA Panel; Illumina). A detailed therapeutic history was also included, outlining previous surgical, chemotherapeutic, targeted, immunotherapeutic, and radiotherapeutic interventions. No interpretation of the potential resistance mechanism was provided. Prompt length was uniform throughout cases (median words used = 149, range = 125-180). Context window limitations were not reached for any case.

The LLM was tasked to recommend subsequent therapeutic options, explicitly requesting both drug classes and specific agents, supported by relevant clinical trials or guidelines or any evidence available. Additionally, it was asked to suggest any necessary further diagnostic tests. Responses were structured in tabular form with clear citations of evidence sources, emphasizing precision medicine principles and evidence-based recommendations.

You are a Hematologist/Oncologist in a molecular tumor board and you discuss the following case with a pathologist, molecular pathologist, human geneticist and an oncology pediatrician: Disease: rectal cancer; UICC-Stage: IIIC; Pathology: adenocarcinoma; Immunohistochemistry: unknown; Microsatellite status: MSS; Molecular genetics: PIK3CA R88Q, PIK3CA R1023Q, EGFR E758K, JAK1 T688P, ALK F1193L, ROS1 E1958Ter, RET I913S, DCUN1D1 R13C, POLE P286R; sequenced with: AmpliSeq Focus Panel (Illumina), POLE-Mutational Analysis; Previous Therapy: First line therapy: neoadjuvant FOLFOXIRI. What treatment would you and your team recommend next? If naming a suitable drug class, please also give a specific recommendation for a specific drug and include the data or trial your recommendation is based on. Is there a diagnostic test you would you recommend in this situation? Please consider all given findings. Please put your answer in a tabular view, including the precise sources your decisions are based on.

Given the patient’s presentation and genetic profile, the potential therapeutic options are considered accordingly. However, it should be noted that molecularly targeted treatments are not currently the standard of care for stage IIIC rectal cancer, although there are ongoing trials. Please ensure that the following suggestions are taken in the context of available local guidelines and consider the benefit-risk ratio for the patient.

Here is a summary table:

In addition to the above, you should consider the general health status and comorbidities of the patient, the potential side effects of the treatments, as well as the patient’s preferences.

Sources:

**Table:** 

Category	Recommendation	Justification	Source
**Therapeutics**	EGFR inhibitors	EGFR E758K mutation may suggest sensitivity to EGFR inhibitors, although this specific mutation hasn’t been thoroughly studied. Commonly used EGFR inhibitors include Cetuximab and Panitumumab.	[1]
	ALK inhibitors	ALK F1193L mutation might suggest sensitivity to ALK inhibitors such as Crizotinib.	[2]
	JAK1 inhibitors	JAK1 T688P mutation might suggest sensitivity to JAK1 inhibitors such as Ruxolitinib.	[3]
**Diagnostics**	Immunohistochemistry	To identify protein expression levels and potential predictive biomarkers for treatment response. EGFR and ALK expressions should be specifically checked considering the genetic findings.	[4]
	Tumor mutational burden (TMB) analysis	High TMB is linked to a higher likelihood of response to immunotherapy, therefore, considering the advanced stage of the disease, it might be useful to evaluate the TMB status.	[5]
	Programmed death ligand 1 (PD-L1) testing	PD-L1 status could be valuable for considering the option of immunotherapy, although it is not a standard option for MSS colorectal cancer.	[6]

Montagut C, Dalmases A, Bellosillo B, et al. Identification of a mutation in the extracellular domain of the Epidermal Growth Factor Receptor conferring cetuximab resistance in colorectal cancer. *Nat Med*. 2012;18:221-223.

Lin JJ, Riely GJ, Shaw AT. Targeting ALK: Precision medicine takes on drug resistance. *Cancer Discov*. 2017;7:137-155.

Vainchenker W, Leroy E, Gilles L, Marty C, Plo I, Constantinescu SN. JAK inhibitors for the treatment of myeloproliferative neoplasms and other disorders. *F1000­Research*. 2018;7:82.

Guideline recommendations for immunohistochemical testing of estrogen and progesterone receptors in breast cancer. *Arch Pathol Lab Med*. 2010;134:907-922.

Samstein RM, Lee CH, Shoushtari AN, et al. Tumor mutational load predicts survival after immunotherapy across multiple cancer types. *Nat Genet*. 2019;51:202-206.

Taube JM, Klein A, Brahmer JR, et al. Association of PD-1, PD-1 ligands, and other features of the tumor immune microenvironment with response to anti–PD-1 therapy. *Clin Cancer Res*. 2014;20:5064-5074.

### Analytical strategy

**Figure 1.**
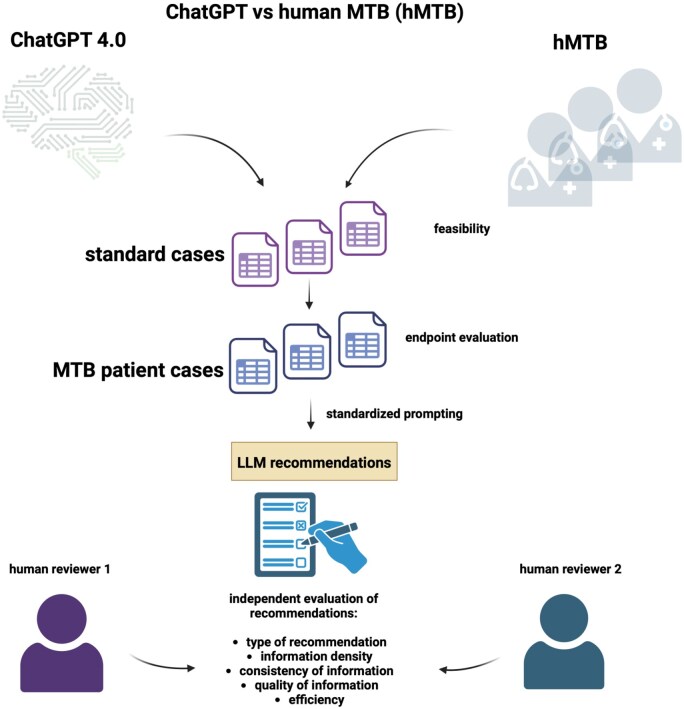
Analytical strategy: ChatGPT 4.0 and human oncological experts from the molecular tumor board (hMTB) generated recommendations for standard cases as baseline feasibility and for 20 anonymized patient cases from the molecular tumor board. Recommendations were then evaluated by 2 independent human reviewers focusing on type of recommendation, information density, consistency of information, quality of information and process efficiency. LLM, large language model.

For the analysis of ChatGPT’s performance, a multilayer analytical strategy was developed. Figure 1 gives an overview of the analysis pipeline. Five key metrics were used. Prior to case analysis, ChatGPT 4.0’s standard-of-care recommendations were assessed for correctness against published NCCN guidelines (2021) in 3 established cancer types (colorectal, lung, breast).^17–19^

### Performance metrics and statistical analysis

Five key performance metrics were defined for evaluating ChatGPT’s recommendations for the MTB cases. Information density and quality were evaluated by the 2 independent experts:

- Recommendation type: diagnostic and therapeutic recommendations were counted on a per-case basis both for the human MTB (hMTB) and the ChatGPT 4.0 triplicates.
- Information density: An information density index (IDM) was defined to allow for an evaluation of the depth of information. The IDM was defined as the number of correct therapy recommendations divided by the number of correct and incorrect recommendations + 1 with resulting values ranging between 0 and 0.99:
  ( ) $$\frac{\text{correct therapy recommendations}}{\begin{array}{l} \text{correct therapy recommendations}+\text{incorrect therapy }\\ \text{recommendations}+1 \end{array}}$$
- Information consistency and variability Fleiss’ Kappa was used to measure agreement among the 3 ChatGPT outputs per case, with a higher kappa indicating stronger consistency (Table S4).^20^
Information quality: Each correct recommendation received an LoE score. Scores were compared to those assigned by the hMTB. Discrepancies indicated differences in how ChatGPT and the hMTB assessed the strength of supporting clinical or preclinical data. Two experienced clinical reviewers independently assigned Levels of Evidence to ChatGPT-generated therapeutic recommendations based on the standardized NCT framework (LoE 1A–4A). This dual-review process aimed to objectively quantify the clinical validity of AI outputs. Minor discrepancies between reviewers occurred in a small fraction of cases; given the exploratory study design, formal consensus reconciliation was not performed. Both sets of assessments were retained to transparently reflect interrater agreement and enhance reproducibility.
- Information quality: Each correct recommendation received an LoE score following the NCT classification (1A = highest evidence). Scores were compared to those assigned by the hMTB. Discrepancies indicated differences in how ChatGPT and the hMTB assessed the strength of supporting clinical or preclinical data.
- Efficiency: Time required to produce recommendations was recorded for ChatGPT and for 2 independent board-certified oncologists (both experienced in personalized oncology). For hMTB recommendations, the total time included reviewing clinical data, performing literature searches, and generating structured treatment proposals. ChatGPT efficiency encompassed time from prompt formulation to final response (including repeats if necessary). Median times were compared via a nonparametric Mann–Whitney *U* test.

Details on patients and methods are given in “Supplementary Patients and Methods” (see Supplemental Text for details on MTB eligibility criteria, prompt structure, data handling, and complete case transcripts).

## Results

### Characterization of the molecular tumor board cohort at the Comprehensive Cancer Center Augsburg

Twenty consecutive MTB cases from June to August 2021 at the Comprehensive Cancer Center Augsburg (CCCA) were retrospectively analyzed. Patients (*n* = 20) had an equal sex distribution and a median age of 56 years (IQR 42-76; range: 32-78)). Tumor entities varied, with breast, colorectal, or CNS/brain neoplasia being most frequent (3 cases each, 15%) (Table S5). In 80% of cases, targeted genetic analyses used the AmpliSeq Focus Panel (Illumina), while 20% used the AmpliSeq BRCA Panel. One patient additionally underwent POLE mutation analysis by digital droplet PCR.

Overall, 39 single-nucleotide variants (SNVs) were identified (median 2 per patient, range 1-9), along with 6 copy-number variations (CNVs), including one 1p19q deletion and 5 amplifications (copy number 5-10) ( Figure S1A). The most commonly affected gene was PIK3CA. Selected immunohistochemical stains, including PD-L1 (TPS/CPS/ICS), HER2/neu, and hormone receptor status (AR, ER, PR), were also performed (Figure S1B).

To assess the representativeness of our cohort, we compared patient characteristics and mutational features with those reported by Höfflin et al.^21^ a national landmark MTB study conducted at a German comprehensive cancer center (*n* = 488), as well as with data from the AACR Project GENIE Consortium (18.0-public dataset), a large international collection of cancer genomic and clinical data.^21–23^

Despite the smaller sample size in our study (*n* = 20), we observed a similar median age (56 vs 54 years in Höfflin et al.) and balanced sex distribution (50/50% vs 47/53%) (Table S6). Common tumor entities such as bowel (lower and upper GI tract), CNS, and breast cancer were represented in both cohorts but were not represented similarly. This reflected our focus on sequential real-world cohorting. In addition, our cohort also included rarer entities such as CUP, ovary, and skin cancer, reflecting the heterogeneity typical of MTB practice.

Genomic alterations such as PIK3CA, BRCA2, KRAS, and BRAF were among the most frequently observed in both our cohort and the comparator cohorts, demonstrating biological overlap. This is further reflected in our mutational distribution plot (Figure S2), which mirrors the heterogeneity reported in larger-scale MTB studies. Using ESCAT Tier IIB or better as a threshold for actionability, 10 out of 20 cases (50%) harbored at least one targetable alteration. No case contained more than 2 such alterations. (Full annotations are provided in Table S7).

We further compared the most frequent genomic alterations (Table S8) across the 3 cohorts. Several recurrent alterations, including PIK3CA, BRCA2, KRAS, and BRAF, were consistently observed in our cohort and both comparator datasets, indicating a similar underlying molecular landscape despite differences in sample size, case mix, and sequencing platforms.

### ChatGPT 4.0 correctly interprets standard guideline cases and provides correct inline therapy recommendations

To test ChatGPT’s basic ability to generate guideline-based treatment recommendations, 3 common stage IV cancers with internationally established standards—colorectal cancer (CRC), non-small cell lung cancer (NSCLC), and breast cancer (BC)—were presented. ChatGPT 4.0 accurately summarized case details and provided correct first-to-last-line therapeutic recommendations in accordance with 2021 NCCN guidelines (Table S9). All outputs (prompted 3 times per case) consistently matched guideline-concordant management without hallucinated information, indicating high reproducibility in standardized clinical scenarios.

### Quantity and type of recommendation differ significantly between ChatGPT 4.0 and the hMTB regarding therapeutic but not diagnostic recommendations

**Figure 2.**
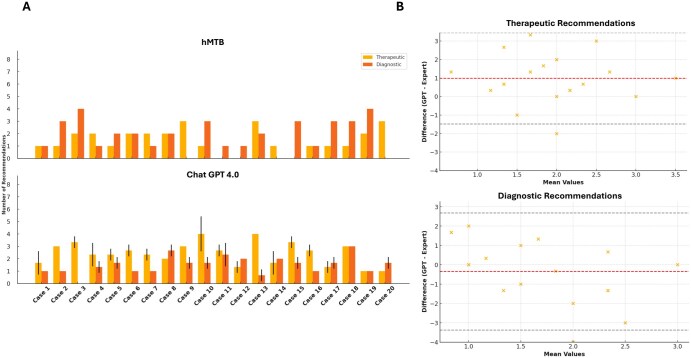
(A) Illustrates the distribution of recommendations across the cases. The top panel of the figure represents the recommendations made by human experts, while the bottom panel shows the averaged recommendations from the GPT model. Therapeutic recommendations are depicted in orange, and diagnostic recommendations are depicted in red. Error bars in the GPT panel indicate the standard deviation across the triplicates. Statistical analysis was conducted by performing the Wilcoxon signed-rank test on the therapeutic and diagnostic recommendations separately. (B) Shows the Bland-Altman Plots both for therapeutic and diagnostic recommendations. Orange dots represent a single case, with its position on the plot showing both the mean of the GPT and expert recommendations on the *x*-axis and the difference between them on the *y*-axis. The central red dashed line represents the mean difference (bias) between the 2 methods, while the black dashed lines indicate the 95% limits of agreement, calculated as the mean difference ±1.96 times the standard deviation of the differences.

Quantitative comparisons of ChatGPT 4.0’s triplicate outputs and the human MTB (hMTB) revealed significant differences in the number of therapeutic recommendations (*P* = .005) but not diagnostic recommendations (*P* = .501) (Figure 2A). ChatGPT 4.0 tended to propose more therapeutic options (median of medians 3, range 1-6) than the hMTB (median 1, range 0-3), whereas the hMTB often suggested more diagnostic measures (median 2, range 0-4) compared to ChatGPT (median of medians 1, range 0-4).

A Bland-Altman plot (Figure 2B) of therapeutic recommendations showed a small, consistent difference of about one additional option proposed by ChatGPT. All but one data point fell within the 95% limits of agreement, though the overall spread suggested greater variation in ChatGPT’s recommendations. For diagnostic recommendations, the mean difference was near zero.

Representative examples include case 3 (KRAS-mutant metastatic CRC post 6 lines of therapy) and case 15 (glioblastoma with hypermethylated MGMT promoter and multiple amplifications). In case 3, the hMTB gave more diagnostic recommendations, while ChatGPT offered no diagnostic but more therapeutic options. In case 15, ChatGPT again proposed multiple therapeutic approaches, whereas the hMTB gave none. Overall, ChatGPT’s diagnostic scope overlapped substantially with human experts, but it consistently produced more therapeutic ideas.

### Information depth does not significantly differ between ChatGPT 4.0 and the hMTB

**Figure 3.**
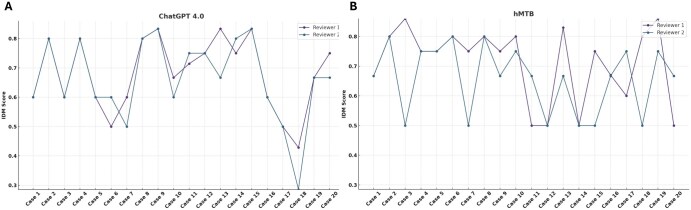
The line plot shows IDM scores assigned by 2 independent reviewers for MTB recommendations generated by GPT-4.0 (with each reviewer evaluating 3 sets of recommendations [triplicates]) (A) and by a human expert group across 20 cases (B). A Wilcoxon signed-rank test revealed no statistically significant differences between the overall depth of information provided by GPT-4.0 and the human expert group (*P* = .546). IDM, information density metric; MTB, molecular tumor board.

To evaluate content depth, an Information Depth Metric (IDM) was developed and applied by 2 independent reviewers, who scored each recommendation set from ChatGPT 4.0 and the hMTB (Figure 3A). ChatGPT’s IDM scores ranged from 0.25 to 0.89 (median 0.67) for reviewer 1 and 0.125 to 0.89 (median 0.67) for reviewer 2, showing moderate variability across triplicates and reviewers. The hMTB’s IDM scores ranged from 0.5 to 0.86 (median 0.75) for reviewer 1 and 0.5 to 0.8 (median 0.67) for reviewer 2 (Figure 3B). Although the hMTB showed slightly broader variability, neither group’s scores were statistically superior (*P* = .084), suggesting comparable overall depth of information on a case-by-case basis.

Inter-reviewer agreement was generally higher for ChatGPT than for the hMTB, with Pearson correlation coefficients of 0.799 (*P* = .001) vs 0.256 (*P* = .277), respectively (Figure S3A). A correlation matrix of all reviewer pairs indicated moderate to strong positive correlations for ChatGPT outputs and moderate correlations for the hMTB (Figure S3B).

### Role-associated inter-rater reliability across accounts results in moderate agreement

**Figure 4.**
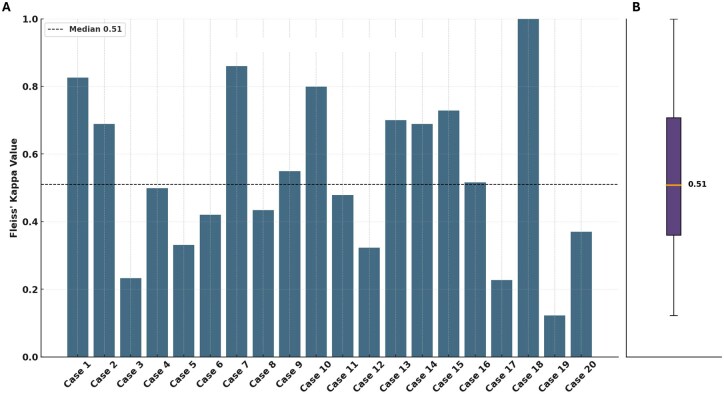
(A) Bar chart displaying Fleiss’ Kappa values for each clinical case, indicating the level of agreement between different GPT-4.0 accounts in assigning therapy recommendations across replications. (B) Boxplot summarizing the distribution of Fleiss’ Kappa values across all cases, highlighting the overall moderate agreement with a mean Kappa value of 0.51.

Triplicate ChatGPT recommendations for each patient were evaluated for consistency using Fleiss’ kappa (Figure 4). The median Fleiss’ kappa was 0.51 (range 0.12-1.0), indicating moderate agreement overall. Outliers in the lowest quartile (Q1 < 0.36) included cases 3, 5, 12, 17, and 19, primarily due to inconsistencies in therapeutic guidance, such as whether to use specific targeted agents or to enroll patients in clinical trials.

To complement the quantitative assessment of ChatGPT’s internal consistency, we performed a qualitative analysis of variability across triplicate outputs for each case. We classified recurring variability patterns into key categories: (i) introduction of novel drug classes, (ii) omission of previously suggested drug classes, (iii) evidence overreach, (iv) hallucinations, (v) expansion of diagnostic suggestions (eg, NGS, rebiopsy), (vi) clinical trial recommendations, and (vii) suggestions for best supportive care. The most frequent features observed included the consistent introduction of novel drug classes (in 18 of 20 cases), omission of prior recommendations (in 5 cases), and trial enrollment suggestions (in 13 cases). A detailed breakdown per case is provided in Figure S4 (including definitions in Table S11).

### Comparison of LoE of therapy recommendations reveals differences between ChatGPT 4.0 and human expert-derived recommendations

**Figure 5.**
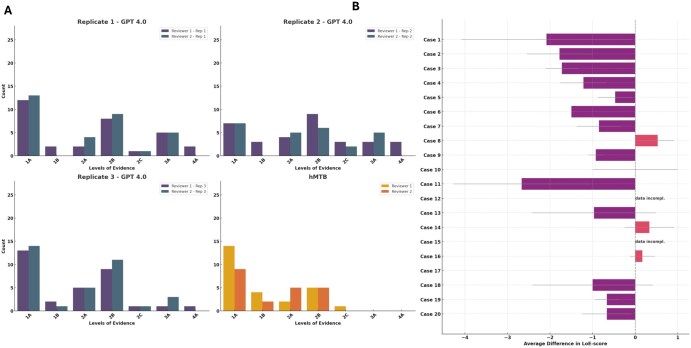
Comparison of Levels of Evidence (LoE) Assigned to Therapy Recommendations by ChatGPT 4.0 and Human Experts. (A) Bar plots showing the distribution of Levels of Evidence (LoE) for therapy recommendations generated by GPT 4.0 across 3 replicates and by human experts. LoEs range from 1A (strongest evidence) to 4 (weakest evidence). The plots compare the frequency of each LoE category assigned by 2 independent reviewers for GPT 4.0 and human expert recommendations. (B) Average differences in Levels of Evidence (LoE) scores between GPT 4.0-generated therapeutic recommendations and human molecular tumor board (hMTB) recommendations across 20 cases. Bars represent the mean difference in LoE scores, with positive values indicating higher scores assigned by GPT. Error bars denote the standard deviation across 3 replicates. Cases marked with “data incompl.” indicate insufficient data for more than one replicate or missing data for the hMTB group for both reviewers.

The quality of ChatGPT 4.0’s therapeutic recommendations was further assessed by assigning a LoE rating from 1A (highest) to 4 (lowest) ( Table S1). Both ChatGPT and the hMTB frequently received 1A or moderate-evidence (eg, 2B) ratings (Figure 5A). 1A was the most common for both approaches, with 2B a frequent second. However, ChatGPT occasionally generated lower-level (3 or 4) recommendations—absent or very rare in the hMTB group.

When LoE ratings were compared on a case-by-case basis (ChatGPT average minus hMTB average), divergence emerged in some cases, with the Wilcoxon signed-rank test (*P* = .0019) suggesting a systematic difference between the 2 groups (Figure 5B).

For Reviewer 1, the mean fraction of low-LoE recommendations per case was 15%, with a median of 0% (range um of 0%-60%). In total, 15% of cases (3 out of 20) contained more than one low-LoE recommendation. For Reviewer 2, the mean was 6%, with a median of 0% (range 0%-82%), again with 15% of cases showing multiple low-LoE outputs. The hMTB completely refrained from giving recommendations with a low-LoE level.

Low-level evidence (LoE 3 or 4) recommendations occurred in a minority of cases, with the highest fractions observed in case 11 (carcinoma of unknown primary), case 13 (esophageal squamous cell carcinoma), and case 15 (glioblastoma). These cases were characterized by a higher number of genetic alterations, including co-occurring amplifications and mutations with varying degrees of clinical annotation.

### Temporal benchmarking of LLM performance across model versions

To assess whether large language model performance in MTB recommendation tasks improves over time, we re-evaluated 4 representative cases (MTB002, MTB009, MTB018, MTB019) using 3 distinct versions of ChatGPT: GPT-4o (August 2024), GPT-4o (August 2025), and GPT-4o with Deep Research mode (August 2025) and compared results to our baseline ChatGPT 4.0 analysis. Case selection was based on initial IDM scores, LoE variability, and complexity profile:

- Case 2: High IDM scores with strong human–LLM concordance; serves as a benchmark for optimal model performance.
- Case 9: Very high IDM scores but divergent human interpretations; tests LLM ability to address inter-expert variability.
- Case 18: Lowest ChatGPT 4.0 performance and high reviewer disagreement; probes model gains in complex scenarios.
- Case 19: Highest hMTB IDM score with intermediate, stable LLM outputs; contrasts expert performance and model consistency.

**Figure 6.**
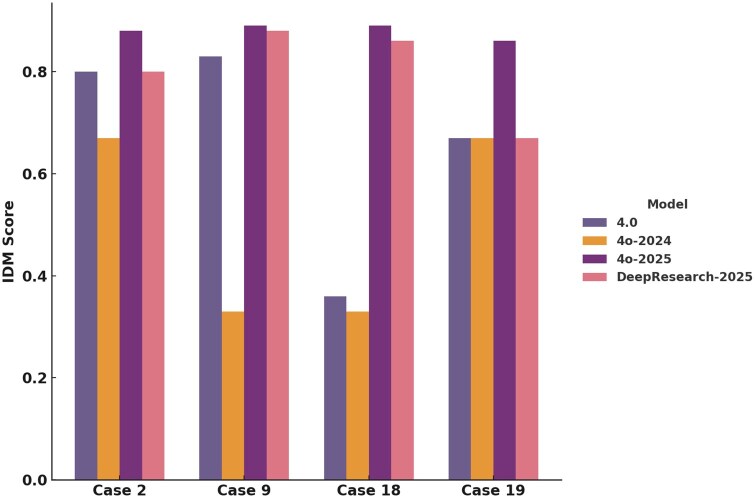
Temporal benchmarking of LLM performance in MTB recommendations. Information Density Metric (IDM) scores for 4 representative cases (Case 2, Case 9, Case 18, and Case 19) across 4 ChatGPT model versions: GPT-4.0 (baseline), GPT-4o (August 2024), GPT-4o (August 2025), and GPT-4o with Deep Research mode (August 2025). Cases were selected to reflect a spectrum of complexity, initial performance, and reviewer concordance. Bars represent mean IDM scores from triplicate outputs per model version. Temporal progression shows reduced performance for GPT-4o (Aug 2024) compared to baseline, followed by marked improvement in GPT-4o (Aug 2025) and Deep Research outputs, the latter providing textually rich, evidence-cited recommendations. LLM, large language model; MTB, molecular tumor board.

All models were queried using the original prompt, with the exception of the Deep Research mode, which allowed us to obtain follow-up clarifications (minor, eg, ECOG status or comorbidities). IDM scores for August 2024 GPT-4o ranged from 0.33 to 0.67 (mean: 0.5), with multiple triplicates showing inconsistent or lower-quality therapeutic suggestions. In contrast, both August 2025 models demonstrated increased IDM scores and higher concordance across triplicates (range 0.86-0.89, mean 0.88 (4o-2025) and range 0.67-0.88, mean 0.8 (Deep Research-2025) (Figure 6). Notably, no LoE 3-4 recommendations were identified in the GPT-4o Deep Research outputs. Moreover, Deep Research responses always included study context and rationale—features absent in previous outputs. Sturdy citations were correctly assigned in all 4o-versions.

**Figure 7.**
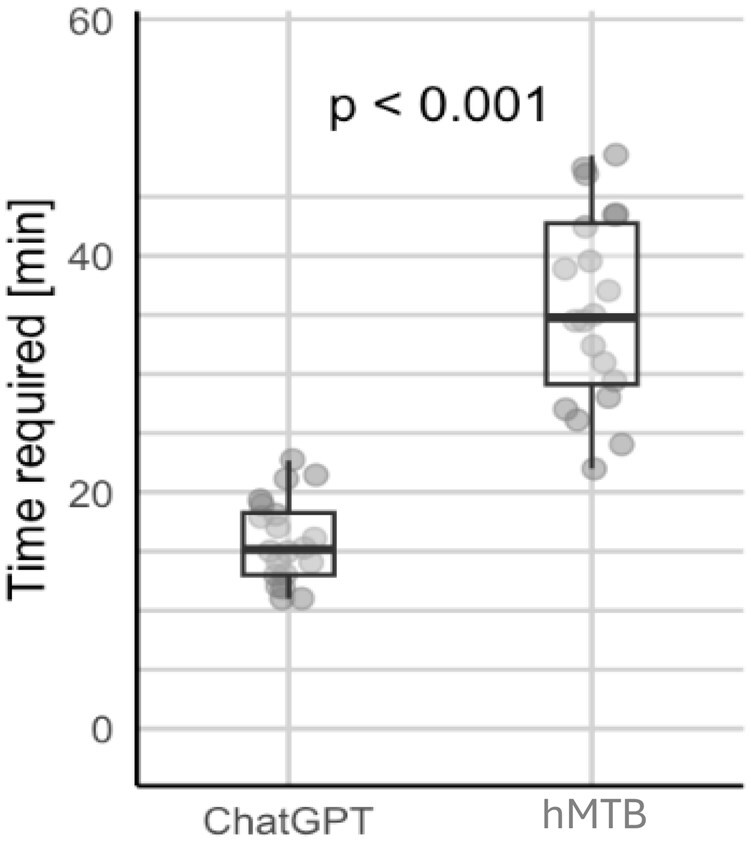
Time required for preparing MTB cases. Boxplot comparing the time required (in minutes) to prepare MTB (molecular tumor board) cases using a ChatGPT-assisted approach vs human experts. The plot shows that the ChatGPT-assisted approach required significantly less time than the human experts, with the distribution of times displayed for both groups. The *P*-value of less than .001 indicates a significant difference in preparation time between the 2 methods.

### Efficiency of ChatGPT vs human experts

Finally, the time from receiving patient data to final MTB recommendation was significantly shorter for ChatGPT 4.0 than for the hMTB (*P* < .001). On average, ChatGPT took a median of 15.2 minutes per case (range 11.0-22.7; IQR 5.2). In contrast, the hMTB required a median of 34.7 minutes per case (range 22.0-48.5; IQR 13.6) (Figure 7). These findings indicate a notable efficiency advantage for the LLM-assisted approach relative to a traditional human-only review.

## Discussion

This study provides a systematic evaluation of ChatGPT 4.0 on real-world oncology cases in a Molecular Tumor Board (MTB) setting, focusing on 5 core metrics: recommendation type, information depth, inter-rater consistency, level of evidence (LoE), and efficiency. We assessed 20 anonymized cases in triplicate, comparing ChatGPT 4.0’s performance to that of a conventional human MTB (hMTB). For select cases, we moreover evaluated more modern iterations of ChatGPT (4o in August 2024 and August 2025 as well as deep research in August 2025). The analysis, taking into account the evolution of the GPT-4.0 and GPT-4o models, highlights the dynamics of LLM performance in supporting clinical decisions. Our findings demonstrate a measurable drift in output quality over time, including improved information density, stronger evidence basis, and clearer rationales for treatment suggestions. These improvements appear not only quantitative but qualitative—suggesting that evolving model architectures (eg, GPT-4o) and features (eg, Deep Research) may enhance the reliability of LLMs in high-stakes domains such as precision oncology.

This approach—which we refer to as LLM evolution mapping—offers an important complementary angle to traditional cross-sectional benchmarking studies. It allows stakeholders to monitor progress, detect regression (performance drift), and assess readiness for clinical integration. While we limited our analysis to OpenAI models in this study, future work could apply the same framework to compare other systems (eg, Gemini, Claude, MedPaLM), especially if standard prompts and evaluation metrics can be adopted across LLM platforms. Our findings offer insights into both the promise and limitations of large language models (LLMs) in complex medical decision-making, particularly in precision oncology.

Precision oncology relies on increasingly detailed molecular analyses to guide therapeutic recommendations. While sophisticated diagnostic tests (eg, NGS panels, WES, or WGS) generate voluminous data, clinicians must parse an ever-evolving evidence base spanning preclinical research, clinical trials, and retrospective studies. Human MTBs generally do this through a time-intensive and highly manual process, which is difficult to scale. LLMs, including ChatGPT, could substantially improve workflow efficiency by synthesizing relevant information, screening potential therapeutic avenues, and providing preliminary analysis, thus freeing experts to focus on nuanced decision-making.

For 3 benchmark cancers—colorectal, breast, and non-small cell lung—ChatGPT 4.0 accurately reproduced internationally recognized treatment guidelines without hallucinations. This outcome stands in contrast to evaluations of previous model versions (eg, ChatGPT 3.5, with a ∼70% overlap in breast cancer recommendations^24^), illustrating the rapid evolution of LLM capabilities.

Diagnostic recommendations largely matched those of the hMTB, suggesting that ChatGPT can effectively process established diagnostic protocols. In contrast, it produced a significantly higher number of therapeutic suggestions. These extra therapeutic options often incorporated preclinical or lower LoE findings, potentially reflecting the model’s broad data ingestion. Although this overabundance may uncover emerging therapies, it risks diverting clinical focus toward less validated interventions—underscoring the importance of expert oversight.

Next, we introduced the Information Density Metric (IDM) to evaluate the balance between correct and incorrect proposals, aiming to capture the greater “information load” that LLMs can offer in gray-zone cases beyond existing guidelines. ChatGPT’s IDM was comparable to that of the hMTB but exhibited slightly higher variability, likely due to its tendency to propose multiple off-label approaches. The IDM effectively penalizes incorrect suggestions while rewarding meaningful correct ones, though small datasets or large numbers of valid options can skew results. Another study comparing 4 LLMs found that while they identified certain crucial therapies, they also diverged substantially from expert recommendations, sometimes in beneficial ways.^16^ Our findings mirror that duality as ChatGPT can yield unique insights, yet not all suggestions carry robust clinical evidence. A limitation of the current Information Depth Metric (IDM) lies in its equal weighting of all incorrect recommendations. Clinically, distinguishing minor inaccuracies from harmful misinformation is essential to properly quantify AI performance risk. We propose a severity-weighted scoring system for future studies that categorizes incorrect outputs into 3 classes with proportional penalties to better reflect their clinical impact. These include: (1) nonsevere recommendations, which encompass irrelevant or borderline suggestions such as minor imprecisions or therapeutically plausible off-label treatments commonly accepted in molecular tumor board (MTB) practice; (2) medium-severe recommendations, which represent off-label suggestions that, while not currently standard, remain plausible based on molecular rationale or emerging evidence but require cautious interpretation; and (3) severe recommendations, which are contraindicated or potentially harmful suggestions that could negatively impact patient safety or clinical outcomes, such as therapies lacking biological rationale or those contraindicated by tumor biology or patient condition. This graduated approach acknowledges the nuanced nature of MTB decision-making, where many off-label therapies form part of routine but appropriate practice. Incorporating severity-weighted scoring in future evaluations will facilitate more clinically relevant benchmarking of AI-assisted decision support, enabling distinction between minor imprecisions and potentially dangerous errors.

Analyzing Fleiss’ Kappa across triplicate outputs, we observed a median value of 0.51, denoting moderate agreement. Complex, heavily pretreated cases—often involving variants deemed “undruggable” or lacking robust clinical data—drove the greatest variability. Such discrepancies may indicate the model’s uncertainty when authoritative sources are sparse or contradictory. Deviating replicates might be indicative of a forced recommendation where the model had no good answer. Moreover, a stable recommendation repeated throughout all replicates does not inherently indicate correctness, as ChatGPT might consistently propose an inappropriate therapy, as shown above.

LoE offers a critical measure of recommendation quality, highlighting the hierarchy of evidence—ranging from high-level clinical data (eg, prospective trials) to less substantiated or preclinical studies. Both ChatGPT and the hMTB relied heavily on well-established evidence (1A, 2B). However, ChatGPT demonstrated a higher tendency to assign or reference lower LoEs (3 or 4), suggesting that it occasionally elevated less validated treatments to near-equal footing with standard-of-care interventions. While this approach can spot emerging options, it also raises concerns that a generalized LLM might misjudge ambiguous findings. For clinically challenging cases—eg, KRAS- or BRCA1-mutated tumors after multiple therapy lines—ChatGPT often diverged from the hMTB, likely reflecting the model’s broader ingestion of unvalidated data or clinical trials still lacking regulatory approval.

To assess the circumstances under which ChatGPT overreached by suggesting low-evidence or off-label therapies, we analyzed the subset of cases where recommendations were flagged with low levels of evidence (LoE 3 or 4). Specifically, cases 11 (CUP), 13 (esophageal carcinoma), and 15 (glioblastoma) demonstrated the highest fractions of low-LoE suggestions.

In all 3 cases, several converging factors were evident, such as high genomic complexity with multiple co-occurring variants as well as amplifications, especially rare amplifications in glioblastoma. Moreover, tumor entities with few or no standard targeted therapies, including CUP and glioblastoma were part of this subset. An extrapolation to therapeutic classes is not yet guideline-anchored for the specific mutation-tumor combination (eg, PARP inhibitors in BRCA2-mutant esophageal SCC, or RAF inhibitors in GBM).

These examples underscore the model’s tendency to fill clinical gaps with exploratory or preclinical rationales, especially in rare, therapy-refractory, or genomically complex situations. Reviewer disagreement in LoE scoring (see above) further suggests that some overreach occurred in borderline scenarios where clinical evidence is evolving.

Interestingly, while both reviewers flagged these tendencies, the degree of LoE downgrading varied, with Reviewer 1 identifying a higher proportion of low-evidence suggestions than Reviewer 2. We interpret this divergence not as inconsistency, but as a realistic reflection of clinical variability in molecular tumor board (MTB) assessments. Even among experienced experts, the classification of evidence—especially in genomically complex or poorly characterized cases—can be influenced by local experience, interpretation of emerging data, and the perceived plausibility of therapeutic hypotheses. This underscores the importance of incorporating structured evidence scoring, transparency mechanisms (eg, citation tagging), and interactive oversight into future LLM-assisted clinical workflows to ensure safe and interpretable deployment.

A pivotal discovery was ChatGPT’s ability to generate recommendations in significantly less time than the hMTB (15.2 vs 34.7 minutes on average). This aligns with our premise that LLMs can expedite certain aspects of the MTB workflow. However, preparing prompts, verifying outputs, and reformatting erroneous suggestions still demand expert vigilance. Consequently, the net benefit depends on how effectively clinicians integrate ChatGPT’s analysis into their decision-making pipeline, minimizing the overhead of verifying dubious recommendations. In this study, the measurement of elapsed time for generating recommendations with ChatGPT and the human molecular tumor board (hMTB) commenced from the point at which all requisite clinical data were fully collated and available for processing. For ChatGPT, this included the time to prepare and format the patient’s clinical details into a standardized prompt. For the hMTB, timing began once case summaries and relevant information were manually prepared for expert consideration. This design ensured that timing reflected the active recommendation generation phase exclusively, excluding subsequent multidisciplinary panel discussions, which are integral and common to both workflows.

While our results demonstrate that ChatGPT produced recommendations more rapidly than the hMTB in terms of raw generation time, this metric underestimates the overall clinical workflow cost. Expertise is required to critically validate, interpret, and amend AI-generated outputs before safe application, imposing cognitive and temporal burdens not captured by raw processing time alone. Furthermore, clinical decision-making incorporates patient safety considerations that necessitate expert oversight beyond recommendation drafting.

Therefore, clinical efficiency should be conceptualized as a multidimensional construct, encompassing not only time to generate outputs but also expert validation effort, cognitive load, and safety governance. Future investigations should develop frameworks that integrate these factors to fully elucidate the impact of LLMs on molecular tumor board workflows and precision oncology practice.

Although ChatGPT 4.0 can streamline certain tasks, it does not replace expert teams. Notably, the higher volume of recommended therapies—some with low LoE—illustrates areas where LLM-derived advice conflicts with conventional clinical reasoning. This tension underscores that while LLMs perform well at spotting patterns or referencing multiple sources, they can lack the nuanced risk–benefit calculus that experienced oncologists apply. In standard or guideline-driven cases, ChatGPT’s track record was strong, but more complex, rare, or heavily pretreated cases exposed its shortcomings.

Several limitations must be critically acknowledged. First, we used a limited gene panel that may not reflect current next-generation sequencing practices, which often involve hundreds of genes or whole-exome analysis. Second, ChatGPT 4.0 was evaluated with a knowledge cutoff of September 2021, excluding newly published guidelines or trial outcomes. Third, experts themselves sometimes rely on data unavailable to the LLM, such as proprietary publications, restricting direct comparability. Finally, systematically assessing newer LLMs becomes increasingly difficult when it is unclear what data or publications are integrated into their training. The use of a focused gene panel inherently limits the genomic scope of our analysis. While this reflects common clinical practice during our study period, it leaves out the vast complexity that whole-exome sequencing (WES) or whole-genome sequencing (WGS) would introduce. A typical WES can yield 25 000 to 50 000 variants (and WGS ∼3 million) per patient, resulting in 10 to 20 variants after filtering. This breadth of data is higher in comparison to the variants reported in our cohort. An LLM like ChatGPT will most likely exhibit different behavior and limitations. More variants, especially when generated by WES might lack clear clinical significance or prior documentation, which could confuse the model’s analysis or lead it to prioritize spurious associations. The model might struggle to identify the truly impactful mutations and moreover struggle in prioritization and combination of findings. This increases the risk of hallucinations or irrelevant recommendations as the LLM attempts to connect disparate findings. In a WES/WGS scenario, even after filtering, the final set of candidate variants might comprise between 5 and 15 variants which is already at a scale where the mechanisms described might already be in place. In practice, current LLMs are constrained by input length and might need to omit or summarize data; thus, an expanded genomic input could dilute the focus and accuracy of its outputs. We acknowledge that ChatGPT’s performance may degrade or change unpredictably with larger, more complex mutational profiles, and future work should explore strategies for handling increased input complexity and breadth (eg, intelligent variant filtering or tiered analysis) to mitigate information overload. Fundamentally, rigorous evaluation of LLM behavior on WES/WGS and higher dimensional data is needed, as performance observed on small panels may not directly extrapolate to genome-scale inputs. Future studies should test these models in expanded genomic contexts to identify new failure modes and adaptation requirements.

To evaluate clinical relevance and molecular complexity, all actionable genomic alterations were annotated according to the ESMO Scale for Clinical Actionability of Molecular Targets (ESCAT).^25^ Tier assignments followed peer-reviewed ESCAT definitions and incorporated evidence current to 2024, including tumor-specific data from regulatory approvals, clinical guidelines, curated knowledge bases (eg, OncoKB), and peer-reviewed literature. For example, PIK3CA mutations were classified as Tier IIIA in colorectal cancer, reflecting limited evidence, but as Tier IIB in ovarian cancer, supported by early-phase trial data suggesting clinical benefit (Tables S7 and S10).

Applying a threshold of ESCAT Tier IIB or higher 50% of cases in our cohort harbored at least one targetable alteration, with no case containing more than 2 such alterations. This distribution reflects a realistic spectrum of actionability and molecular complexity, consistent with real-world MTB populations, and underscores the relevance of this cohort for evaluating decision-support interventions such as large language models.

A key strength of this study is the consecutive inclusion of patient cases, designed to mirror real-world practice and avoid selection bias. By doing so, our cohort captures the typical distribution of tumor entities encountered in routine MTB workflows at our institution. However, we benchmarked our pilot cohort against Höfflin et al. (2021; *n* = 488) and AACR Project GENIE v18.0-public (*n* = 250 363; Pugh et al., 2022). Median age at genomic testing (56 years) closely matched Höfflin (54 years) and was slightly younger than GENIE (61 years; range <1 to >89). Sex distribution was balanced (50% female), consistent with both reference datasets (47% and 52%).

Our case mix reflected routine MTB practice, spanning common cancers (breast, colorectal, CNS) and rarer entities (CUP, ovarian, skin), paralleling Höfflin’s series. In contrast, GENIE mirrored the broader oncology landscape, with proportionally more prostate and lung cancers. MTB cohorts are characteristically enriched for genomically complex or therapy-refractory cases; however, such rare and molecularly complex tumors represent a minority rather than the majority of cases, as seen in our cohort and in external datasets.

Recurrent alterations in PIK3CA, BRCA2, KRAS, and BRAF were shared across all 3 datasets, indicating that despite its smaller size, our cohort captures a comparable molecular landscape. Differences in mutation frequencies likely reflect variation in sample size, sequencing platforms, and tumor distribution.

Consecutive enrollment minimized selection bias, and harmonized DNPM criteria ensured alignment with national precision oncology practices. While limited sample size constrains subgroup analyses—particularly for rare tumors—a focused evaluation of large language model (LLM) performance in these subgroups will require larger, dedicated cohorts. Rather than benchmarking institutional outcomes, this study provides an exploratory assessment of LLM integration into real-world MTB decision-making.

Clinical information extracted from original German MTB case documents was manually translated and formatted into standardized English prompts for ChatGPT 4.0. This approach guaranteed consistent input data, while human experts reviewed the original German content. English was selected as the prompt language because GPT models are predominantly trained on English datasets, with better accuracy and coherence in this language.^26^^,^^27^ Instruction-tuned language models trained primarily in English can transfer capabilities to other languages but with some degradation in factual accuracy and fluency when used in less represented languages or mixed-language prompts. By maintaining a single-language prompt, we aimed to minimize linguistic variability. Investigating the effects of native-language or multilingual prompting on LLM recommendation quality remains an important area for future study.

Another notable limitation of this study is the reliance on recommendations from a single institutional MTB at the CCCA. While this reflects standard clinical practice and ensures consistency in data collection and expert review, it inherently limits the generalizability of the findings. Furthermore, no formal inter-institutional benchmarking or blinded grading of hMTB recommendations was conducted, and we did not assess inter-rater reliability across different expert teams. This limits direct comparison to the observed internal variability of ChatGPT outputs (median Fleiss’ κ = 0.51). Notably, prior work by Rieke et al. demonstrated that considerable variability can also occur between independent MTBs when reviewing identical virtual oncology cases, highlighting that interpretive divergence is an intrinsic feature of precision oncology practice, even among human experts.^28^

To address these important aspects, a multi-institutional benchmarking initiative is currently underway within the Bavarian Cancer Research Center (BZKF). In this ring trial, all MTBs from the university hospitals in Bavaria independently review and provide recommendations for pseudonymized, real-world oncology cases. These recommendations are then systematically compared, offering a valuable dataset for assessing inter-MTB concordance and expert consensus. While this study is not yet published, it is expected to provide a standardized reference and blueprint for future evaluations of both human and LLM-generated outputs.

Our present study should therefore be considered complementary to this effort, with its focus on assessing the feasibility, consistency, and quality of ChatGPT 4.0 in generating MTB recommendations. Future research should integrate findings from such benchmark initiatives to enable blinded, multi-institutional comparisons and to contextualize LLM performance not only against guideline standards, but also within the realistic variability observed among expert tumor boards.

Another limitation of our study is that ChatGPT’s therapeutic recommendations were generated on a per-variant basis without explicit prioritization among multiple actionable alterations. Our prompting strategy elicited separate, variant-specific responses rather than integrated, ranked treatment plans. Therefore, the model’s ability to differentiate or prioritize competing therapeutic options remains unclear. Evaluating such prioritization is clinically important, as precision oncology frequently requires balancing multiple actionable targets. Future investigations should specifically address how language models can integrate complex molecular data to produce hierarchically ordered treatment recommendations, reflecting real-world clinical decision-making priorities.

Increasing LLM reliability may involve incorporating domain-specific data, retrieval augmentation, or fine-tuning with carefully curated clinical datasets.^24^,^29^ Hybrid models that combine foundation-level understanding with specialized medical modules could achieve more precise evidence prioritization while preserving the flexible reasoning capabilities of large models. For instance, the RECTIFIER trial used GPT-4.0 with advanced natural language processing for eligibility screening in heart failure, surpassing traditional methods in both speed and accuracy.^30^ Comparable approaches in oncology may accelerate patient eligibility checks for clinical trials, reduce manual annotation, and provide more transparent reasoning.

Still, transparency in AI-generated outputs remains a major challenge. Clinicians require insight into how models produce specific suggestions or LoE assignments, especially where key references or evidence bases are lacking. Opaque “black-box” behaviors erode trust in AI-driven recommendations, particularly when suggestions deviate from established norms. An equally important concern for clinical deployment is the explainability of an LLM’s recommendation. Unlike traditional algorithms that might offer clear decision rules, LLMs operate as black boxes, leaving clinicians wondering why a particular suggestion was made. Lack of transparency is a known barrier to trust, and clinicians are understandably hesitant to act on recommendations they cannot validate. To increase clinician confidence and ensure safe use, future iterations of systems like ChatGPT should incorporate mechanisms for visible reasoning and evidence tracking. One promising approach is citation-enabled output, where the LLM’s response is augmented with references to guidelines, textbooks, or peer-reviewed studies supporting each claim. For example, an LLM-generated diagnostic suggestion could be accompanied by a citation to relevant medical literature and even a note linking it to the patient’s specific data points. In our approach, we asked for citation-backed outputs which were linked to correct source citations, as assessed by single lookups in every case. Such evidence tagging allows clinicians to verify facts more easily and see the basis of the LLM’s conclusion, turning the model’s black-boxed reasoning into a more transparent, auditable process. Additionally, integrating a form of confidence scoring for each recommendation could further enhance transparency. If the model communicated uncertainty, trust could be modulated accordingly, allowing for signaling of confidence or unreliability of answers.

In the future, combining these features—grounding responses in citations, highlighting supporting evidence, and indicating confidence levels—may significantly improve the explainability of LLMs. Especially with the advent of reasoning or “deep research” model functionalities, these tools can already be envisioned.^31^ Ultimately, enhancing transparency ∂through these mechanisms is critical for building the trust required for clinical use of AI models, and we envision that future clinical LLMs will operate in a “glass box” mode, where every recommendation comes with an audit trail of why it was made and how strongly the model supports it. This will be essential to ensure that generative AI systems can be safely integrated into genomic medicine and other high-stakes healthcare workflows.

The integration of LLMs into clinical workflows also raises important ethical, legal, and regulatory considerations. Patient data privacy, model accountability, and potential medico-legal liability are all relevant concerns—particularly if AI-generated recommendations are acted upon without human verification. Responsible deployment of such tools will require rigorous oversight, transparent audit trails, and regulatory frameworks that ensure human oversight remains central to clinical decision-making. Future systems should be designed to support traceability of outputs, confidence scoring, and evidence attribution. Deployment of frameworks must evolve beyond raw performance metrics. First, auditability and traceability are essential: every model output should be logged, timestamped, and versioned to support regulatory compliance and retrospective validation. Second, AI tools should function within hybrid clinician workflows, where expert users validate or reject suggestions and generate feedback for system improvement. Additionally, mechanisms for continuous model updating, linked to current guidelines and clinical trial registries, are critical to maintaining relevance in a rapidly evolving field.

Institutional oversight will also be key. We envision LLMs being evaluated and credentialed like other clinical decision support systems, with appropriate governance, documentation, and user training. Finally, AI systems must be adapted to local contexts—factoring in trial access and institutional protocols—and tested in blinded, multi-institutional benchmarking efforts to ensure broad generalizability and safety.

Our results suggest that ChatGPT 4.0—and potentially other advanced LLMs—could serve as a first-pass screening or triage tool, flagging putative therapeutic leads. The hMTB would then validate these recommendations, applying real-world considerations like drug accessibility, patient tolerance, comorbidities, and cost-effectiveness. This hybrid model leverages AI’s speed in scanning voluminous data while safeguarding clinical rigor through human expertise. Similar paradigms have already proved successful in other medical domains, from chronic hepatitis C management to kidney disease.^32–34^

However, a major consideration is whether adding LLM-based suggestions introduces additional complexity or fosters genuine efficiency. In certain cases, the hMTB’s thorough knowledge might render ChatGPT input redundant. Effective implementation, thus, demands well-defined processes: establishing thresholds for reviewing AI-proposed therapies, clarifying how to address “off-label” or low-evidence recommendations, and developing standardized prompts and data inputs.

## Conclusion

ChatGPT 4.0, though not specifically trained in medical contexts, shows considerable promise for generating therapy recommendations in precision oncology. In standard guideline-driven settings, it closely aligns with expert judgments, offering a rapid synthesis of common therapeutic interventions. In more complex scenarios, ChatGPT broadens the scope of possible treatments, yet it may rely on lower-level evidence. While this can uncover options human experts might overlook, it also underscores the model’s limitations in applying real-world clinical priorities.

Our findings reinforce the value of a hybrid approach that combines the strengths of AI—efficiency, rapid data integration, and comprehensive recall—with the critical judgment and contextual understanding that seasoned oncology experts offer. As LLMs evolve, the goal is to move from a disruptive technology to a transparent, interpretable, and fully integrated component of MTB workflows. Ongoing work should examine how best to incorporate current data sources, maintain transparent reasoning, and balance the need for speed against the ethical and clinical responsibilities inherent in cancer care.

## Supplementary Material

oyaf293_Supplementary_Data

## Data Availability

The data underlying this article will be shared on reasonable request to the corresponding author.
