# Supplementary material for: Large language model processing capabilities of ChatGPT 4.0 to generate molecular tumor board recommendations—a critical evaluation on real world data
Source: Oncologist. 2025 Sep 18;30(10):oyaf293. doi: 10.1093/oncolo/oyaf293 (PMC12557318; doi:10.1093/oncolo/oyaf293)
Supplement: oyaf293_Supplementary_Data [file oyaf293_supplementary_data.zip › Supplemental Table 1.docx]

**Supplemental Table 1 – Levels of evidence (LoE)^1^**

| **1A** | Drug is approved for the same tumor type harboring the specific biomarker. | *Crizotinib in NSCLC with EML4-ALK fusion14* |
| --- | --- | --- |
| **1B** | Predictive value of the biomarker or clinical effectiveness of the corresponding drug in a molecularly stratified cohort was demonstrated in an adequately powered prospective study or a meta-analysis. | *Erlotinib in NSCLC with EGFR amplification15, 16* |
| **2A** | Predictive value of the biomarker or clinical effectiveness of the drug in a molecularly stratified cohort was demonstrated in a prospective trial with biomarkers as a secondary objective or an adequately powered retrospective cohort or case-control study in the same tumor type. | [*Vemurafenib in NSCLC with BRAF V600E mutation4*](https://onlinelibrary.wiley.com/doi/10.1002/ijc.30828#ijc30828-bib-0004) |
| **2B** | Predictive value of the biomarker or clinical effectiveness of the drug in a molecularly stratified cohort was demonstrated by clinical data in a different tumor type. | [*Everolimus in NSCLC with PTEN loss-of-function mutation, loss, or deletion17*](https://onlinelibrary.wiley.com/doi/10.1002/ijc.30828%22%20/l%20%22ijc30828-bib-0017) |
| **2C** | Case study or single unusual responder indicates the biomarker is associated with response to the drug, supported by scientific rationale. | *Sorafenib in NSCLC with BRAF G469V/R mutation18, 19* |
| **3** | Preclinical data (*in vitro* or *in vivo* models and functional genomics) demonstrate that the biomarker predicts response of cells to drug treatment. | *Dasatinib in NSCLC with DDR2 mutation20* |
| **4** | Biological rationale exists that links the drug to the altered signaling pathway or relevant basket. No reported clinical or preclinical data on the response to the drug. | *Panobinostat in NSCLC with SMARCA4 loss-of-function mutation* |

1 Horak, P. *et al.* Precision oncology based on omics data: The NCT Heidelberg experience. *Int J Cancer* **141**, 877-886 (2017). <https://doi.org:10.1002/ijc.30828>
