# Supplementary material for: Large language model processing capabilities of ChatGPT 4.0 to generate molecular tumor board recommendations—a critical evaluation on real world data
Source: Oncologist. 2025 Sep 18;30(10):oyaf293. doi: 10.1093/oncolo/oyaf293 (PMC12557318; doi:10.1093/oncolo/oyaf293)
Supplement: oyaf293_Supplementary_Data [file oyaf293_supplementary_data.zip › Supplemental Table 2.docx]

**Supplemental Table 2 - Prompt overview**

Case 1

You are a Hematologist / Oncologist in a molecular tumorboard and you discuss the following case with a pathologist, molecular pathologist, human geneticist and an oncology paediatrician: Disease: Breast Cancer; UICC-Stage: IV; Pathology: Invasive lobular carcinoma; Immunohistochemistry: ER IRS 6/12, PR IRS 0/12, AR IRS 12/12, HER2/neu: 1, TPS 1%, CPS 1, ICS < 1 ; Microsatellitestatus: MSS; Molecular genetics : PIK3CA H1047L; sequenced with :Ampliseq Focus Panel (Illumina) Previous Therapy: First Line Therapy: Surgery, adjuvant Radiochemotherapy; Second-line: Tamoxifen; Third-line: Eribulin; Fourth-line: Fulvestrant, Fifth-line: Nab-Paclitaxel; sixth-line: Palbociclib / Fulvestrant, seventh-line: Letrozol, Eigth-line: Alpelisib/Fulvestrant,ninth-line: Carboplatin / liposomal Doxorubicin.What treatment would you and your team recommend next? If naming a suitable drug class, please also give a specific recommendation for a specific drug and include the data or trial your recommendation is based on. Is there a diagnostic test you would you recommend in this situation? Please consider all given findings. Put your answer please in a table, including the precise sources your decisions are based on.

Case 2

You are a Hematologist / Oncologist in a molecular tumorboard and you discuss the following case with a pathologist, molecular pathologist, human geneticist and an oncology paediatrician: Disease: Cervical Cancer; UICC-Stage: IV; Pathology: adenocarcinoma; Immunohistochemistry: TPS 30%, CPS 35, ICS 3%, HPV-18 positive; Microsatellitestatus: MSS; Molecular genetics : MAP2K1 F53L, ALK R672S; sequenced with :Ampliseq Focus Panel (Illumina) Previous Therapy: First Line Therapy: Surgery, Second Line Therapy: Surgery with neoadjuvant systemtherapy, Third Line Therapy: Carboplatin/Taxol, Fourth Line Therapy: Radiatio.What treatment would you and your team recommend next? If naming a suitable drug class, please also give a specific recommendation for a specific drug and include the data or trial your recommendation is based on. Is there a diagnostic test you would you recommend in this situation? Please consider all given findings. Put your answer please in a table, including the precise sources your decisions are based on.

Case 3

You are a Hematologist / Oncologist in a molecular tumorboard and you discuss the following case with a pathologist, molecular pathologist, human geneticist and an oncology paediatrician: Disease: caecum cancer; UICC-Stage: IV; Pathology: adenocarcinoma low grade; Immunohistochemistry: unknown; Microsatellitestatus: MSS; Molecular genetics : KRAS G12D, PIK3CA H1047R; sequenced with :Ampliseq Focus Panel (Illumina) Previous Therapy: First Line Therapy: surgery, Second line therapy: Capecitabin + Oxaliplatin, Third line therapy: surgery, fourth line therapy: folinic acid + fluorouracil + irinotecan + bevacizumab, fifth line therapy: folinic acid + fluorouracil + oxaliplatin + bevacizumab, sixth line therapy: folinic acid + fluorouracil + irinotecan + bevacizumab.What treatment would you and your team recommend next? If naming a suitable drug class, please also give a specific recommendation for a specific drug and include the data or trial your recommendation is based on. Is there a diagnostic test you would you recommend in this situation? Please consider all given findings. Put your answer please in a table, including the precise sources your decisions are based on.

Case 4

You are a Hematologist / Oncologist in a molecular tumorboard and you discuss the following case with a pathologist, molecular pathologist, human geneticist and an oncology paediatrician: Disease: breast cancer; UICC-Stage: IV; Pathology: Invasive breast cancer (NST) ; Immunohistochemistry: ER IRS 12/12, PR IRS 12/12, HER2/neu: 0, Ki-67: 90%, Typ Luminal B; Microsatellitestatus: unknown; Molecular genetics : Amplification in the MYC gene with a copy number of 5; sequenced with :Ampliseq Focus Panel (Illumina) Previous Therapy: First line therapy: surgery + tamoxifen, second line therapy: surgery, third line therapy: radiatio + local hyperthermia, fourth line therapy: exemestane + everolimus, fifth line therapy: CDK4/6 inhibitor + exemestane.What treatment would you and your team recommend next? If naming a suitable drug class, please also give a specific recommendation for a specific drug and include the data or trial your recommendation is based on. Is there a diagnostic test you would you recommend in this situation? Please consider all given findings. Put your answer please in a table, including the precise sources your decisions are based on.

Case 5

You are a Hematologist / Oncologist in a molecular tumorboard and you discuss the following case with a pathologist, molecular pathologist, human geneticist and an oncology paediatrician: Disease: breast cancer; UICC-Stage: IV; Pathology: Invasive breast cancer (NST) ; Immunohistochemistry: ER IRS 12/12, PR IRS 12/12, Her2/neu: 0, Ki-67: 30%; Microsatellitestatus: unknown; Molecular genetics : BRCA2 K1691AfsTer15, ESR1 D538G; sequenced with :Ampliseq BRCA Panel (Illumina); all exonic and flancing introns of BRCA1 and BRCA2; Ampliseq Focus Panel (Illumina) Previous Therapy: First line therapy: zoledronic acid + palbociclib + letrozole, Second line therapy: zoledronic acid + palbociclib + fulvestrant, third line therapy: exemestane + everolimus.What treatment would you and your team recommend next? If naming a suitable drug class, please also give a specific recommendation for a specific drug and include the data or trial your recommendation is based on. Is there a diagnostic test you would you recommend in this situation? Please consider all given findings. Put your answer please in a table, including the precise sources your decisions are based on.

Case 6

You are a Hematologist / Oncologist in a molecular tumorboard and you discuss the following case with a pathologist, molecular pathologist, human geneticist and an oncology paediatrician: Disease: cholangiocellular cancer; UICC-Stage: IV; Pathology: adenocarcinoma; Immunohistochemistry: TPS < 1%, CPS < 1, ICS < 1; Microsatellitestatus: MSS; Molecular genetics : IDH2 R172G, MET Y71C; sequenced with :Ampliseq Focus Panel (Illumina) Previous Therapy: First Line therapy: gemcitabine + cisplatin, second line therapy: folinic acid + 5-fluorouracil + irinotecan, third line therapy: carboplatin + paclitaxel.What treatment would you and your team recommend next? If naming a suitable drug class, please also give a specific recommendation for a specific drug and include the data or trial your recommendation is based on. Is there a diagnostic test you would you recommend in this situation? Please consider all given findings. Put your answer please in a table, including the precise sources your decisions are based on.

Case 7

You are a Hematologist / Oncologist in a molecular tumorboard and you discuss the following case with a pathologist, molecular pathologist, human geneticist and an oncology paediatrician: Disease: ovarian cancer; UICC-Stage: IV; Pathology: clear cell ovarian carcinoma; Immunohistochemistry: unknown; Microsatellitestatus: MSS; Molecular genetics : PIK3CA C420R, ERBB2 R633Q, BRCA2 K3326*; sequenced with :Ampliseq BRCA Panel (Illumina); all exonic and flancing introns of BRCA1 and BRCA2; Ampliseq Focus Panel (Illumina) Previous Therapy: First line therapy: paclitaxel + carboplatin, second line therapy: Doxorubicin hydrochloride + bevacizumab, third line therapy: paclitaxel + carboplatin, fourth line therapy: topotecan, fifth line therapy: gemcitabine.What treatment would you and your team recommend next? If naming a suitable drug class, please also give a specific recommendation for a specific drug and include the data or trial your recommendation is based on. Is there a diagnostic test you would you recommend in this situation? Please consider all given findings. Put your answer please in a table, including the precise sources your decisions are based on.

Case 8

You are a Hematologist / Oncologist in a molecular tumorboard and you discuss the following case with a pathologist, molecular pathologist, human geneticist and an oncology paediatrician: Disease: cholangiocellular cancer, CUP-syndrome; UICC-Stage: IV; Pathology: adenocarcinoma; Immunohistochemistry: Pos: cytokeratin 19, SATB2 in sections, in < 5% of cells CK20; Neg: CA 19-9, TTF1, CDx2; Microsatellitestatus: MSS; Molecular genetics : IDH1 R132C; sequenced with :Ampliseq Focus Panel (Illumina) Previous Therapy: First line therapy: cisplatin + gemcitabine.What treatment would you and your team recommend next? If naming a suitable drug class, please also give a specific recommendation for a specific drug and include the data or trial your recommendation is based on. Is there a diagnostic test you would you recommend in this situation? Please consider all given findings. Put your answer please in a table, including the precise sources your decisions are based on.

Case 9

You are a Hematologist / Oncologist in a molecular tumorboard and you discuss the following case with a pathologist, molecular pathologist, human geneticist and an oncology paediatrician: Disease: Glioblastoma multiforme (IDH-mutant); UICC-Stage: IV; Pathology: Glioblastoma multiforme, Grade 4; Immunohistochemistry: pos: GFAP, MAP2, p53, IDH1; Ki-67: 30%: neg: ATRX; Microsatellitestatus: unknown; Molecular genetics : IDH1 R132H, PIK3CA C420R, MGMT hypermethylated; del(1p19q); sequenced with :Ampliseq Focus Panel (Illumina) Previous Therapy: First line therapy: surgery.What treatment would you and your team recommend next? If naming a suitable drug class, please also give a specific recommendation for a specific drug and include the data or trial your recommendation is based on. Is there a diagnostic test you would you recommend in this situation? Please consider all given findings. Put your answer please in a table, including the precise sources your decisions are based on.

Case 10

You are a Hematologist / Oncologist in a molecular tumorboard and you discuss the following case with a pathologist, molecular pathologist, human geneticist and an oncology paediatrician: Disease: Glioblastoma multiforme; UICC-Stage: IV; Pathology: partly spindle cell and giant cell containing glioblastoma multiforme; Immunohistochemistry: pos: GFAP, OLIG2, p53, ATRX; Ki-67: 60%: neg: IDH1; Microsatellitestatus: unknown; Molecular genetics : PIK3CA P104L, unmethylated MGMT promoter; sequenced with :Ampliseq Focus Panel (Illumina) Previous Therapy: First line therapy: radiation plus temozolomide, second line therapy: Lomustine.What treatment would you and your team recommend next? If naming a suitable drug class, please also give a specific recommendation for a specific drug and include the data or trial your recommendation is based on. Is there a diagnostic test you would you recommend in this situation? Please consider all given findings. Put your answer please in a table, including the precise sources your decisions are based on.

Case 11

You are a Hematologist / Oncologist in a molecular tumorboard and you discuss the following case with a pathologist, molecular pathologist, human geneticist and an oncology paediatrician: Disease: carcinoma of unkown primary (CUP); UICC-Stage: IV; Pathology: adenocarcinoma; Immunohistochemistry: pos.: CK7, CDX2; neg.: Glypican, CD30; Microsatellitestatus: MSS; Molecular genetics : PIK3CA E365K, MYC N26S; sequenced with :Ampliseq Focus Panel (Illumina) Previous Therapy: First line therapy: cisplatin + gemcitabine.What treatment would you and your team recommend next? If naming a suitable drug class, please also give a specific recommendation for a specific drug and include the data or trial your recommendation is based on. Is there a diagnostic test you would you recommend in this situation? Please consider all given findings. Put your answer please in a table, including the precise sources your decisions are based on.

Case 12

You are a Hematologist / Oncologist in a molecular tumorboard and you discuss the following case with a pathologist, molecular pathologist, human geneticist and an oncology paediatrician: Disease: rectal cancer; UICC-Stage: IV; Pathology: adenocarcinoma; Immunohistochemistry: Her2/neu: negative; Microsatellitestatus: MSS; Molecular genetics : KRAS Q61H, PIK3CA E545K; sequenced with :Ampliseq Focus Panel (Illumina) Previous Therapy: First line therapy: neoadjuvant R-CTx with irradiation of the tumor region + Capecitabine, second line therapy: surgery + FOLFOX, third line therapy: radiation + fluorouracil, fourth line therapy: folinic acid + fluorouracil + irinotecan + bevacizumab, fifth line therapy: fluorouracil + bevacizumab, sixth line therapy: radiation + fluorouracil.What treatment would you and your team recommend next? If naming a suitable drug class, please also give a specific recommendation for a specific drug and include the data or trial your recommendation is based on. Is there a diagnostic test you would you recommend in this situation? Please consider all given findings. Put your answer please in a table, including the precise sources your decisions are based on.

Case 13

You are a Hematologist / Oncologist in a molecular tumorboard and you discuss the following case with a pathologist, molecular pathologist, human geneticist and an oncology paediatrician: Disease: esophagus cancer; UICC-Stage: IV; Pathology: Squamous cell carcinoma; Immunohistochemistry: TPS = 2%, CPS = 6, ICS > 1, FISH NTRK neg.; Microsatellitestatus: unknown; Molecular genetics : BRCA2 L1908R, ATM P292L, TP53, ASXL1 R676E, FAT1 Y3949, CCND1-amplification (> 10 copies), FGF19-amplification (> 10 copies), FGF3-amplification (> 10 copies), FGF4-amplification (> 10 copies),; sequenced with :Ampliseq BRCA Panel (Illumina); all exonic and flancing introns of BRCA1 and BRCA2; Ampliseq Focus Panel (Illumina) Previous Therapy: First line therapy: cisplatin + fluorouracil, second line therapy: carboplatin + fluorouracil.What treatment would you and your team recommend next? If naming a suitable drug class, please also give a specific recommendation for a specific drug and include the data or trial your recommendation is based on. Is there a diagnostic test you would you recommend in this situation? Please consider all given findings. Put your answer please in a table, including the precise sources your decisions are based on.

Case 14

You are a Hematologist / Oncologist in a molecular tumorboard and you discuss the following case with a pathologist, molecular pathologist, human geneticist and an oncology paediatrician: Disease: osteosarcoma; UICC-Stage: III; Pathology: osteosarcoma; Immunohistochemistry: unknown; Microsatellitestatus: unknown; Molecular genetics : PIK3CA H1047R; sequenced with :Ampliseq Focus Panel (Illumina) Previous Therapy: First line therapy: surgery + radiation surgery.What treatment would you and your team recommend next? If naming a suitable drug class, please also give a specific recommendation for a specific drug and include the data or trial your recommendation is based on. Is there a diagnostic test you would you recommend in this situation? Please consider all given findings. Put your answer please in a table, including the precise sources your decisions are based on.

Case 15

You are a Hematologist / Oncologist in a molecular tumorboard and you discuss the following case with a pathologist, molecular pathologist, human geneticist and an oncology paediatrician: Disease: Glioblastoma multiforme; UICC-Stage: IV; Pathology: Glioblastoma multiforme, Grade 4; Immunohistochemistry: NTRK negativ; Microsatellitestatus: MSS; Molecular genetics : hypermethylated MGMT-promoter, RAF1-amplification (4.5 copies), CTNNB1-amplification (4.5 copies), PIK3CA-amplification (4.5 copies), DCUN1D1-amplification (4.5 copies)
RAF1, CTNNB1, PIK3CA, and DCUN1D1, each with a copy number of 4.5; sequenced with :Ampliseq Focus Panel (Illumina) Previous Therapy: First line therapy: radiotherapy + Temozolomide followed by surgery.What treatment would you and your team recommend next? If naming a suitable drug class, please also give a specific recommendation for a specific drug and include the data or trial your recommendation is based on. Is there a diagnostic test you would you recommend in this situation? Please consider all given findings. Put your answer please in a table, including the precise sources your decisions are based on.

Case 16

You are a Hematologist / Oncologist in a molecular tumorboard and you discuss the following case with a pathologist, molecular pathologist, human geneticist and an oncology paediatrician: Disease: Salivary gland duct cancer; UICC-Stage: IV; Pathology: carcinoma; Immunohistochemistry: TPS: 0%, CPS = 40, ICS = 24%; Microsatellitestatus: Her2/neu: 3+ at the beginning, in recurrence after acqusition of tissue: Her2/neu: negative, AR positive; Molecular genetics : BRAF I592N; sequenced with :Ampliseq Focus Panel (Illumina) Previous Therapy: First line therapy: surgery + radiotherapy, second line therapy: Pertuzumab + Trastuzumab + Docetaxel, third line therapy: Pertuzumab + Trastuzumab + Docetaxel, fourth line therapy: leuprorelin acetate + Bicalutamide.What treatment would you and your team recommend next? If naming a suitable drug class, please also give a specific recommendation for a specific drug and include the data or trial your recommendation is based on. Is there a diagnostic test you would you recommend in this situation? Please consider all given findings. Put your answer please in a table, including the precise sources your decisions are based on.

Case 17

You are a Hematologist / Oncologist in a molecular tumorboard and you discuss the following case with a pathologist, molecular pathologist, human geneticist and an oncology paediatrician: Disease: prostate cancer; UICC-Stage: IV; Pathology: adenocarcinoma; Immunohistochemistry: TPS: 1%, CPS: >1, ICS: < 1%; Microsatellitestatus: unknown; Molecular genetics : BRAF K601E; sequenced with :Ampliseq Focus Panel (Illumina) Previous Therapy: First line therapy: surgery, second line therapy: Atezolizumab.What treatment would you and your team recommend next? If naming a suitable drug class, please also give a specific recommendation for a specific drug and include the data or trial your recommendation is based on. Is there a diagnostic test you would you recommend in this situation? Please consider all given findings. Put your answer please in a table, including the precise sources your decisions are based on.

Case 18

You are a Hematologist / Oncologist in a molecular tumorboard and you discuss the following case with a pathologist, molecular pathologist, human geneticist and an oncology paediatrician: Disease: rectal cancer; UICC-Stage: IIIC; Pathology: adenocarcinoma; Immunohistochemistry: unknown; Microsatellitestatus: MSS; Molecular genetics : PIK3CA R88Q, PIK3CA R1023Q, EGFR E758K, JAK1 T688P, ALK F1193L, ROS1 E1958Ter, RET I913S, DCUN1D1 R13C, POLE P286R; sequenced with :Ampliseq Focus Panel (Illumina), POLE-Mutational Analysis Previous Therapy: First line therapy: neoadjuvant FOLFOXIRI.What treatment would you and your team recommend next? If naming a suitable drug class, please also give a specific recommendation for a specific drug and include the data or trial your recommendation is based on. Is there a diagnostic test you would you recommend in this situation? Please consider all given findings. Put your answer please in a table, including the precise sources your decisions are based on.

Case 19

You are a Hematologist / Oncologist in a molecular tumorboard and you discuss the following case with a pathologist, molecular pathologist, human geneticist and an oncology paediatrician: Disease: NSCLC; UICC-Stage: IV; Pathology: adenocarcinoma; Immunohistochemistry: TPS: 0, CPS: 0, ICS: 0; Ki-67: 70%; Microsatellitestatus: unknown; Molecular genetics : ERBB2 Y772_A775dup; sequenced with :Ampliseq Focus Panel (Illumina) Previous Therapy: First line therapy: Pembrolizumab, Pemetrexed, Carboplatin; Second line therapy: Docetaxel, Ramucirumab; Third line therapy: Carboplatin, Vinorelbin.What treatment would you and your team recommend next? If naming a suitable drug class, please also give a specific recommendation for a specific drug and include the data or trial your recommendation is based on. Is there a diagnostic test you would you recommend in this situation? Please consider all given findings. Put your answer please in a table, including the precise sources your decisions are based on.

Case 20

You are a Hematologist / Oncologist in a molecular tumorboard and you discuss the following case with a pathologist, molecular pathologist, human geneticist and an oncology paediatrician: Disease: melanoma; UICC-Stage: IV; Pathology: melanoma; Immunohistochemistry: pos: SOX10; Microsatellitestatus: unknown; Molecular genetics : NRAS Q61R; sequenced with :Ampliseq Focus Panel (Illumina) Previous Therapy: First line therapy: Ipilimumab + Nivolumab.What treatment would you and your team recommend next? If naming a suitable drug class, please also give a specific recommendation for a specific drug and include the data or trial your recommendation is based on. Is there a diagnostic test you would you recommend in this situation? Please consider all given findings. Put your answer please in a table, including the precise sources your decisions are based on.
