# Supplementary material for: Large language model processing capabilities of ChatGPT 4.0 to generate molecular tumor board recommendations—a critical evaluation on real world data
Source: Oncologist. 2025 Sep 18;30(10):oyaf293. doi: 10.1093/oncolo/oyaf293 (PMC12557318; doi:10.1093/oncolo/oyaf293)
Supplement: oyaf293_Supplementary_Data [file oyaf293_supplementary_data.zip › Supplemental Table 4.docx]

**Supplemental Table 4**

| **Kappa < 0** | **Poor agreement** |
| --- | --- |
| **Kappa 0.0 – 0.2** | **Slight agreement** |
| **Kappa 0.21 – 0.4** | **Fair agreement** |
| **Kappa 0.41 – 0.6** | **Moderate agreement** |
| **Kappa 0.61 – 0.8** | **Substantial agreement** |
| **Kappa 0.81 – 1.00** | **Almost perfect agreement** |

Interpretation of Fleiss‘ Kappa

Recommendations considered synonymous are as follows:

- clinical trial = study
- comprehensive genomic profiling = NGS = liquid biopsy
- drugs of respective classes are considered as synonymous recommendation
- single chemotherapy compounds are considered equal after second line therapy
