# Supplementary material for: Large language model processing capabilities of ChatGPT 4.0 to generate molecular tumor board recommendations—a critical evaluation on real world data
Source: Oncologist. 2025 Sep 18;30(10):oyaf293. doi: 10.1093/oncolo/oyaf293 (PMC12557318; doi:10.1093/oncolo/oyaf293)
Supplement: oyaf293_Supplementary_Data [file oyaf293_supplementary_data.zip › Supplemental Table 9.docx]

**Supplemental Table 9 – Prompts and Answers for Standard Cases**

**Case List**

1. Left-sided colorectal cancer (**CRC**), UICC stage IV, microsatellite stable (MSS), *BRAF* wildtype (WT), *RAS* mutated (mut) (p.G12A)
2. Non-small cell lung cancer (**NSCLC**), UICC stage IV, programmed cell death ligand-1 (PD-L1): TPS 15% (TC 0), CPS 15%, ICS < 1 (IC 0), Exon 19 deletion (del): EGFR p.E746_A750del
3. Breast Cancer (**BC**), (invasive-lobular breast cancer), UICC stage IV, human epidermal growth factor receptor 2 (Her2/NEU) negative, estrogen receptor (ER) 12/12, progesteron receptor (PR) 12/12, MSS

| **Case** | **Therapy Line** | **Recommendation** |
| --- | --- | --- |
| **CRC – 1^st^ replicate** | **First** | **FOLFOX or FOLFIRI + Bevacizumab** |
| **CRC – 1^st^ replicate** | **First** | **Combination chemotherapy (FOLFOX or FOLFIRI) with the potential addition of Bevacizumab (VEGF inhibitor).** |
| **CRC – 1^st^ replicate** | **First** | **Combination chemotherapy: Regimens such as FOLFOX (folinic acid, fluorouracil, oxaliplatin) or FOLFIRI (folinic acid, fluorouracil, irinotecan). Inclusion of bevacizumab, a VEGF inhibitor, can often be added to enhance efficacy.** |
| **CRC – 2^nd^ replicate** | **Second** | **Switch to the alternative regimen (e.g., switch from FOLFOX to FOLFIRI or vice versa) and continued bevacizumab, or consideration of other targeted therapies like aflibercept or ramucirumab** |
| **CRC – 2^nd^ replicate** | **Second** | **If FOLFOX was used first, FOLFIRI could be used now, and vice versa. Bevacizumab might also be considered if not previously used. Other options may include Regorafenib and TAS-102 (Trifluridine/Tipiracil)** |
| **CRC – 2^nd^ replicate** | **Second** | **If the first-line regimen was FOLFOX, FOLFIRI might be used, and vice versa. Additional treatment options might include aflibercept or ramucirumab (VEGF inhibitors), or regorafenib or TAS-102 (trifluridine/tipiracil), depending on what was used in the first-line** |
| **CRC – 3^rd^ replicate** | **Third** | **Regorafenib or trifluridine/tipiracil (Lonsurf)** |
| **CRC – 3^rd^ replicate** | **Third** | **If not used in second-line treatment, Regorafenib and TAS-102 (Trifluridine/Tipiracil) could be considered at this stage. Participation in clinical trials may also be explored** |
| **CRC – 3^rd^ replicate** | **Third** | **If second-line therapies prove ineffective, third-line treatment may include regorafenib or TAS-102 if not used previously. Consideration for participation in clinical trials for experimental therapies, dependent on the patient's overall health status and preferences.** |
| **BC – 1^st^ replicate** | **First** | **- Endocrine therapy: Aromatase inhibitors (letrozole, anastrozole for post-menopausal) or fulvestrant (for pre-menopausal with ovarian function suppression), or tamoxifen (for pre-menopausal) <br> - CDK4/6 inhibitor: Palbociclib, ribociclib, or abemaciclib added to endocrine therapy <br> - Chemotherapy: if disease is significantly symptomatic and patient's general health is good** |
| **BC – 1^st^ replicate** | **First** | **Aromatase Inhibitors (letrozole, anastrozole) or Fulvestrant, in combination with a CDK4/6 inhibitor (palbociclib, ribociclib, abemaciclib)** |
| **BC – 1^st^ replicate** | **First** | **1. Hormonal Therapy (e.g. aromatase inhibitors like letrozole, anastrozole, or exemestane; tamoxifen; fulvestrant) <br> 2. CDK4/6 Inhibitors (e.g. palbociclib, ribociclib, abemaciclib) often used in combination with hormonal therapy <br> 3. mTOR Inhibitors (e.g. everolimus) potentially used with hormonal therapy** |
| **BC – 2^nd^ replicate** | **Second** | **- Alternative endocrine therapy: switch from aromatase inhibitor to fulvestrant, or vice versa <br> - mTOR inhibitor: Everolimus combined with endocrine therapy <br> - Chemotherapy: taxanes, anthracyclines, or capecitabine** |
| **BC – 2^nd^ replicate** | **Second** | **Switch from AI to Fulvestrant or vice versa, possibly in combination with a different CDK4/6 inhibitor not used in the first line OR Everolimus in combination with exemestane** |
| **BC – 2^nd^ replicate** | **Second** | **1. Change in Hormonal Therapy (switching to a different medication if the cancer progresses on one form of hormonal therapy) <br> 2. Chemotherapy (e.g. anthracyclines like doxorubicin; taxanes like paclitaxel; others like capecitabine or eribulin)** |
| **BC – 3^rd^ replicate** | **Third** | **- Additional chemotherapy: other chemotherapeutic agents not yet used <br> - Endocrine therapy: if not tried yet, another type of endocrine therapy <br> - Investigational therapies: consider enrolling in clinical trials for new therapies or drug combinations <br> - Supportive care: managing symptoms and side effects, maintaining quality of life** |
| **BC – 3^rd^ replicate** | **Third** | **Chemotherapy options (taxanes, anthracyclines if not used previously, capecitabine, eribulin, or others), Ovarian suppression (if premenopausal), Consideration of clinical trials** |
| **BC – 3^rd^ replicate** | **Third** | **Alternate Hormonal Therapy or Chemotherapy (further switches in hormonal therapy or chemotherapy) <br> 2. Investigational Therapies or Clinical Trials** |
| **NSCLC – 1^st^ replicate** | **First** | **EGFR-TKIs: Osimertinib, Erlotinib, Gefitinib, Afatinib** |
| **NSCLC – 1^st^ replicate** | **First** | **EGFR Tyrosine Kinase Inhibitors (TKIs): Osimertinib** |
| **NSCLC – 1^st^ replicate** | **First** | **EGFR Tyrosine Kinase Inhibitors (TKIs): osimertinib, gefitinib, erlotinib, or afatinib** |
| **NSCLC – 2^nd^ replicate** | **Second** | **- Osimertinib (if not used as first-line and T790M mutation develops) <br> - Platinum-based chemotherapy (e.g., Carboplatin with Pemetrexed) <br> - PD-1/PD-L1 inhibitors (e.g., Pembrolizumab, Nivolumab, Atezolizumab) (limited effectiveness due to low PD-L1 expression)** |
| **NSCLC – 2^nd^ replicate** | **Second** | **1. Chemotherapy: Combination of a platinum-based drug (like cisplatin or carboplatin) with another chemotherapy agent such as pemetrexed. 2. Next Generation TKIs (if the patient develops resistance to first-line TKIs, usually via a T790M mutation).** |
| **NSCLC – 2^nd^ replicate** | **Second** | **- If T790M mutation emerges, consider osimertinib (if not used in first-line treatment) <br> - If no T790M mutation or previously treated with osimertinib, consider chemotherapy with or without anti-angiogenesis inhibitors or immunotherapy** |
| **NSCLC – 3^rd^ replicate** | **Third** | **- Switch to different chemotherapy regimen <br> - Other classes of targeted therapy (e.g., VEGF inhibitors like Bevacizumab or ALK inhibitors if there's an ALK rearrangement) <br> - Clinical trials <br> - PD-1/PD-L1 inhibitor (if not used previously and other options have been exhausted)** |
| **NSCLC – 3^rd^ replicate** | **Third** | **1. Immunotherapy: Pembrolizumab, atezolizumab, nivolumab. Efficacy may be limited based on the relatively low PD-L1 expression. 2. Chemotherapy or Combination Therapy: Other chemotherapy drugs or combinations thereof might be employed. Also, a combination of chemotherapy and immunotherapy could be considered.** |
| **NSCLC – 3^rd^ replicate** | **Third** | **- Immune checkpoint inhibitor (if not used in second-line therapy and if PD-L1 expression is ≥1%) <br> - Docetaxel chemotherapy possibly in combination with the angiogenesis inhibitor ramucirumab <br> - Consideration of clinical trials** |
