# Supplementary material for: Large language model processing capabilities of ChatGPT 4.0 to generate molecular tumor board recommendations—a critical evaluation on real world data
Source: Oncologist. 2025 Sep 18;30(10):oyaf293. doi: 10.1093/oncolo/oyaf293 (PMC12557318; doi:10.1093/oncolo/oyaf293)
Supplement: oyaf293_Supplementary_Data [file oyaf293_supplementary_data.zip › Supplemental_Figures_Merged.pptx]

## Slide 1
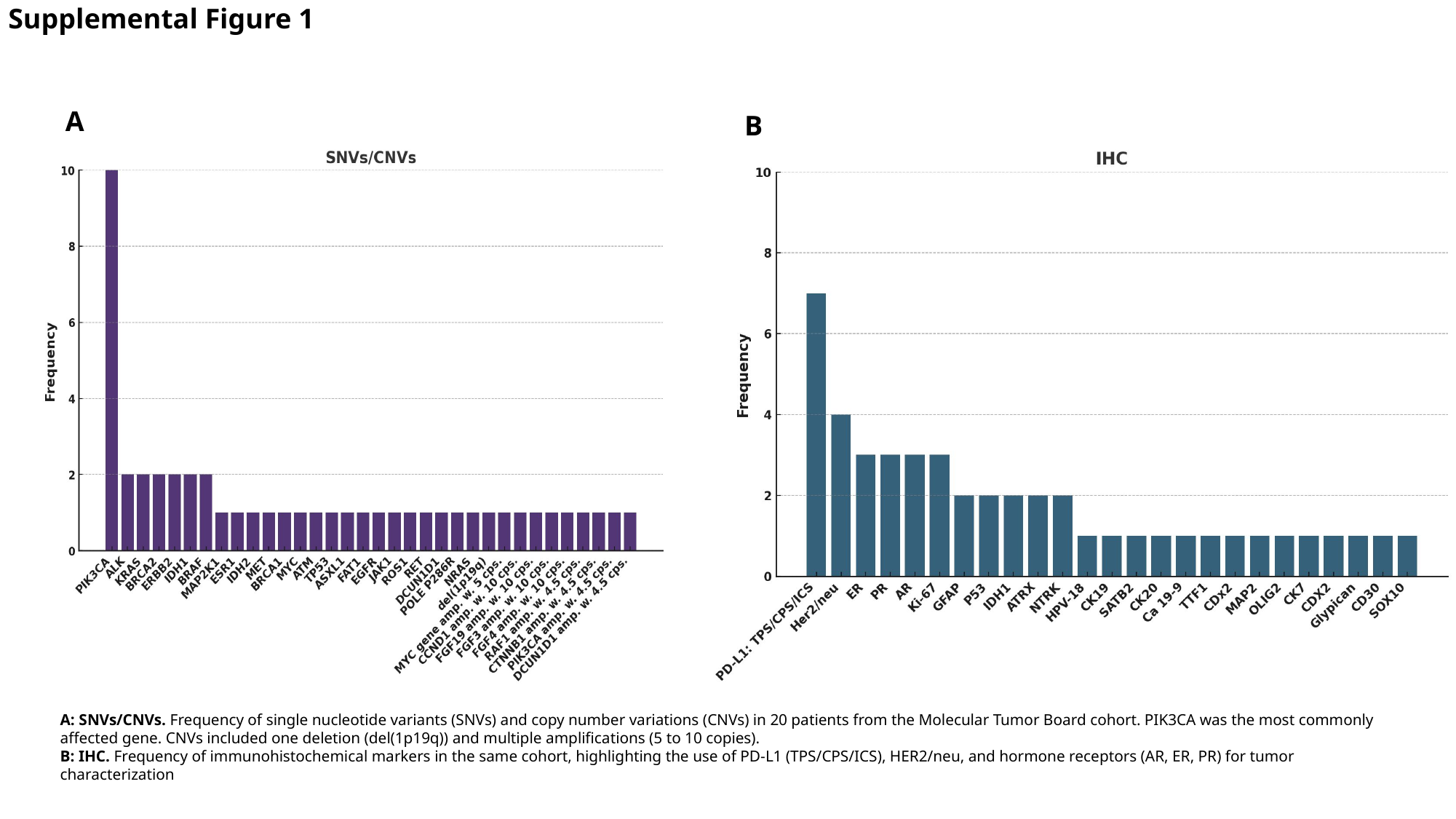

Supplemental Figure 1
A
B
A: SNVs/CNVs. Frequency of single nucleotide variants (SNVs) and copy number variations (CNVs) in 20 patients from the Molecular Tumor Board cohort. PIK3CA was the most commonly affected gene. CNVs included one deletion (del(1p19q)) and multiple amplifications (5 to 10 copies).
B: IHC. Frequency of immunohistochemical markers in the same cohort, highlighting the use of PD-L1 (TPS/CPS/ICS), HER2/neu, and hormone receptors (AR, ER, PR) for tumor characterization

## Slide 2
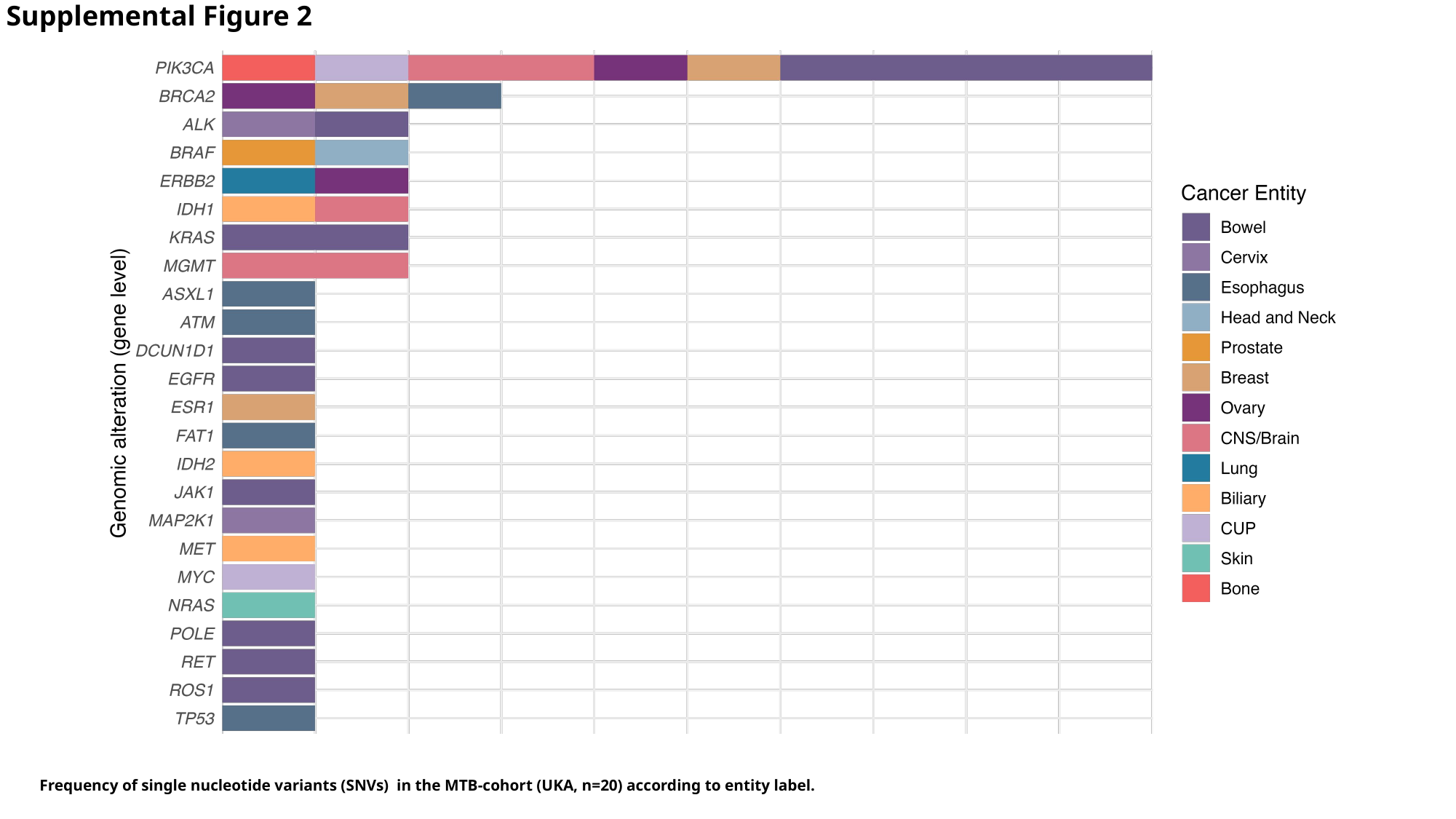

Supplemental Figure 2
Frequency of single nucleotide variants (SNVs) in the MTB-cohort (UKA, n=20) according to entity label.

## Slide 3
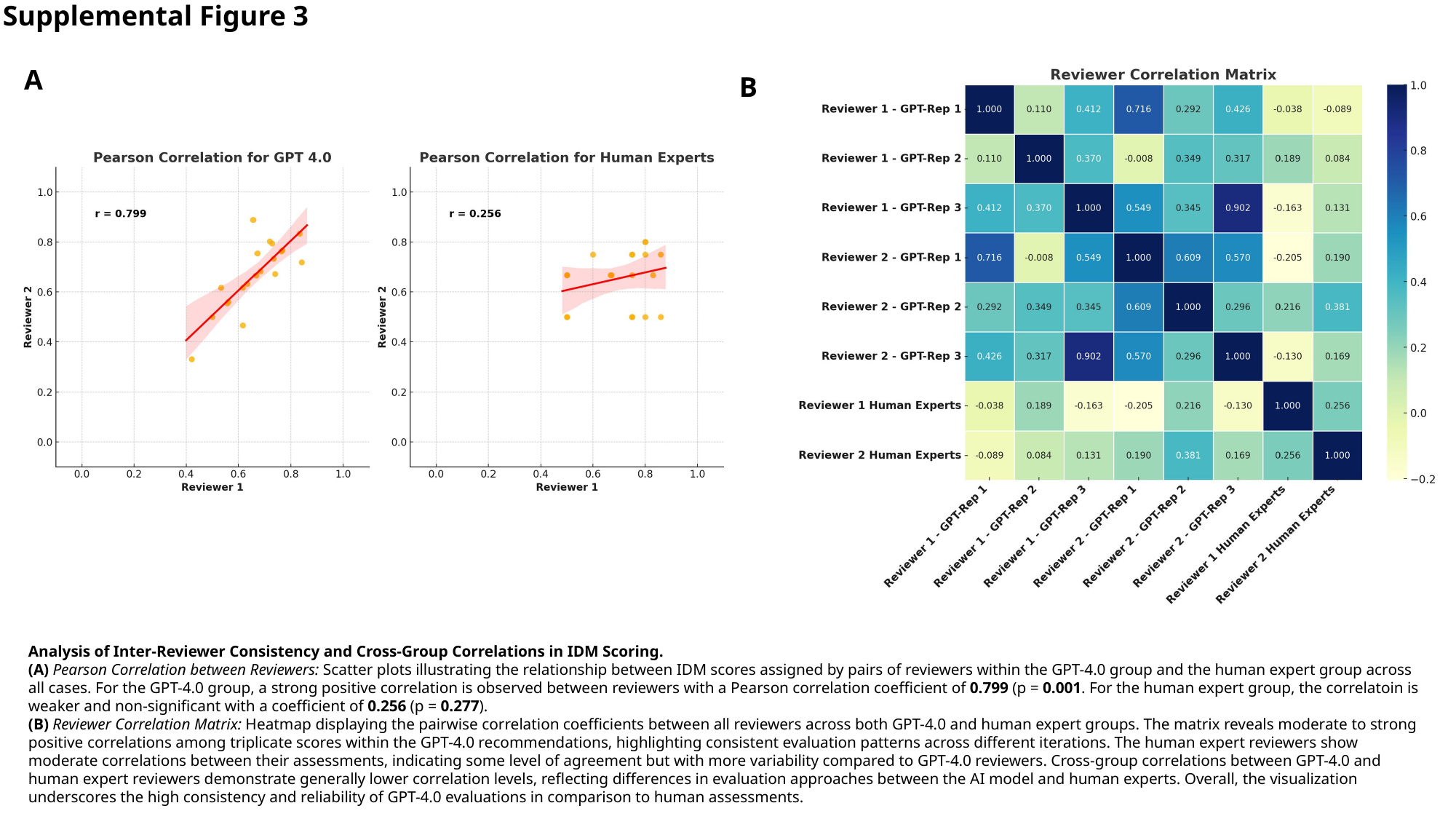

Supplemental Figure 3
A
B
Analysis of Inter-Reviewer Consistency and Cross-Group Correlations in IDM Scoring.
(A) Pearson Correlation between Reviewers: Scatter plots illustrating the relationship between IDM scores assigned by pairs of reviewers within the GPT-4.0 group and the human expert group across all cases. For the GPT-4.0 group, a strong positive correlation is observed between reviewers with a Pearson correlation coefficient of 0.799 (p = 0.001. For the human expert group, the correlatoin is weaker and non-significant with a coefficient of 0.256 (p = 0.277).
(B) Reviewer Correlation Matrix: Heatmap displaying the pairwise correlation coefficients between all reviewers across both GPT-4.0 and human expert groups. The matrix reveals moderate to strong positive correlations among triplicate scores within the GPT-4.0 recommendations, highlighting consistent evaluation patterns across different iterations. The human expert reviewers show moderate correlations between their assessments, indicating some level of agreement but with more variability compared to GPT-4.0 reviewers. Cross-group correlations between GPT-4.0 and human expert reviewers demonstrate generally lower correlation levels, reflecting differences in evaluation approaches between the AI model and human experts. Overall, the visualization underscores the high consistency and reliability of GPT-4.0 evaluations in comparison to human assessments.

## Slide 4
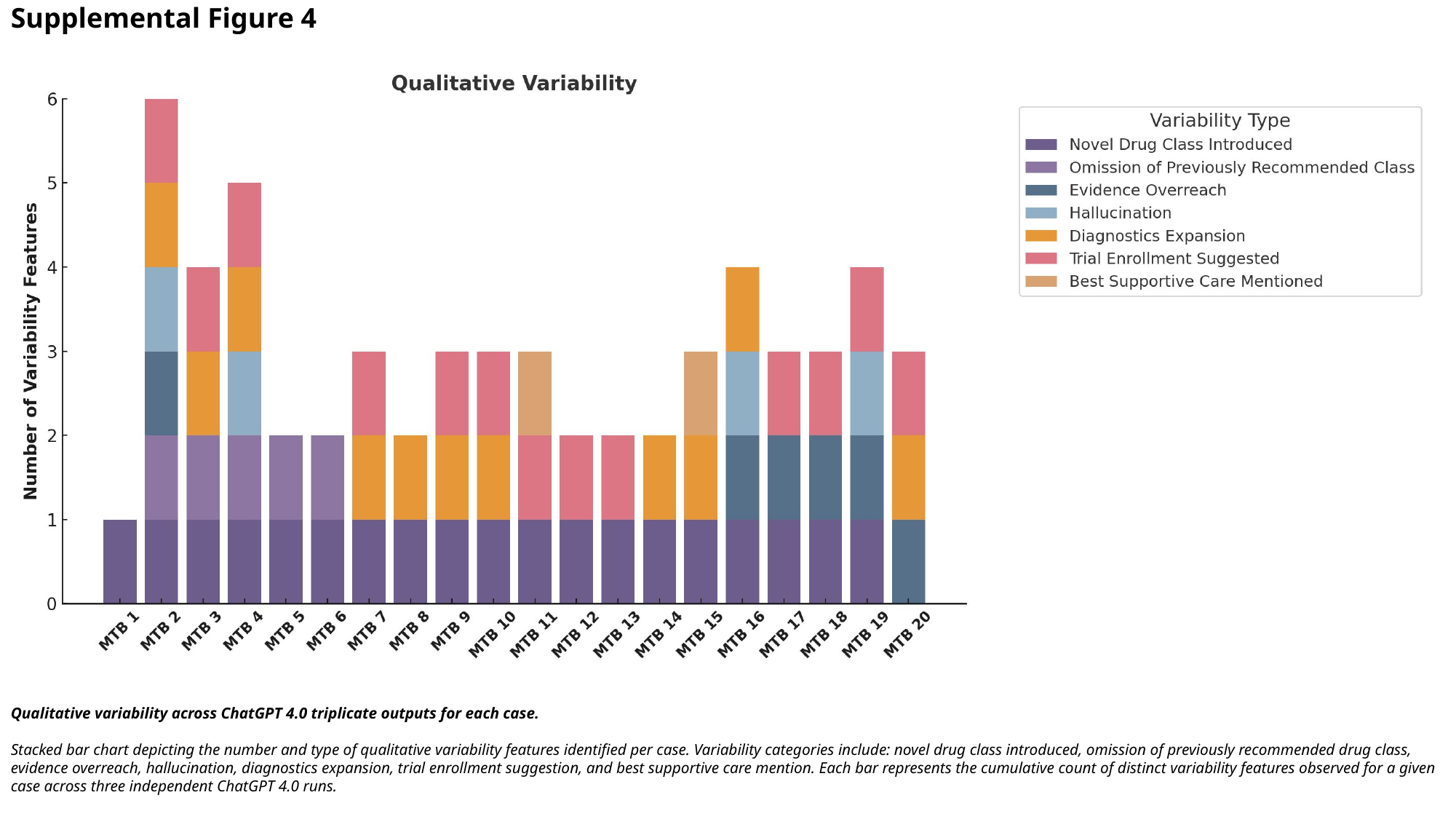

Supplemental Figure 4
Qualitative variability across ChatGPT 4.0 triplicate outputs for each case.
Stacked bar chart depicting the number and type of qualitative variability features identified per case. Variability categories include: novel drug class introduced, omission of previously recommended drug class, evidence overreach, hallucination, diagnostics expansion, trial enrollment suggestion, and best supportive care mention. Each bar represents the cumulative count of distinct variability features observed for a given case across three independent ChatGPT 4.0 runs.
