# Supplementary material for: Large language model processing capabilities of ChatGPT 4.0 to generate molecular tumor board recommendations—a critical evaluation on real world data
Source: Oncologist. 2025 Sep 18;30(10):oyaf293. doi: 10.1093/oncolo/oyaf293 (PMC12557318; doi:10.1093/oncolo/oyaf293)
Supplement: oyaf293_Supplementary_Data [file oyaf293_supplementary_data.zip › Supplemental_Patients_and_Methods_UPDATED_v2.docx]

**Supplemental Patients and Methods**

*Molecular Tumor Board at the Comprehensive Cancer Center Augsburg*

The MTB at the Comprehensive Cancer Center Augsburg (CCCA) was founded in 2018 as an interdisciplinary facility with the aim of improving the individual treatment outcomes of oncology patients for whom approved therapies are ineffective or who suffer from rare cancer entities. At the MTB-CCCA a team of specialists from oncology, pathology, molecular pathology, human genetics and bioinformatics discusses individual cases on a weekly basis. Relevant patient inclusion criteria for the MTB-CCCA are 1) consent, 2) exhaustion of guideline-compliant or state-of-the-art therapies or foreseeable exhaustion of appropriate therapies, 3) patients with tumor diseases without established treatment options, 4) patients with diagnostic challenges (e. g. carcinoma of unknown primary), 5) rare tumors, 6) unusual disease courses and 7) life expectancy and condition of the patient that make a continuation of therapy justifiable. A structured diagnostic and/or therapeutic recommendation including both clinical and preclinical evidence for an identified biomarker is given based on database and literature review. All recommendations follow the NCT classification for levels of evidence (LoE)11. Details are given in **Supplemental Table 1**. MTB recommendations are accompanied by summaries of the relevant literature or clinical studies and can be used ad hoc for reimbursement applications to healthcare providers.

*Patients and Patient Data*

This analysis was conducted prior to the extension of the ChatGPT 4.0 knowledge cut-off date in November 2023. Therefore, the selection of MTB cases and the cut-off date of the data for this analysis was based on the previous ChatGPT 4.0 knowledge cut-off date in September 2021. Thus, "real" sequential MTB cases from June to August 2021 were included and reviewed in the analysis. 20 consecutive cases with proven genetic aberrations and a structured report with comments, LoE and consecutive treatment recommendations were included in the analysis. For these MTB cases, aggregated patient data were presented to the interdisciplinary MTB team on PowerPoint slides. The minimum required data set included the diagnosis; the stage (as defined by the Union for International Cancer Control [UICC]); the histology (i.e., morphology determined according to the latest revision of the International Classification of Diseases for Oncology [ICD-O], 3rd edition, 1st revision [World Health Organization, 2020]); the biopsy site; available immunohistochemical staining; and all previous lines of therapy, including the best response to therapy and the duration of that response. In these patients, molecular genetic analyses were performed on available tissue biopsies using multi-gene NGS panels (AmpliSeq for Illumina Cancer HotSpot Panel v2, AmpliSeq for Illumina Focus Panel, AmpliSeq for Illumina BRCA Panel and Agilent SureMASTER HRR Panel). Aberrations were defined either by the identification of a single nucleotide variant (SNV), by copy number variations (CNVs) or by fusions. Immunohistochemical staining of proteins serving as antibody targets (e.g. antibody-drug conjugates) was performed when required. Gender, age, and most pre-existing comorbidities were not considered, as treatment recommendations were made independently of these factors. The final treatment recommendation was at the discretion of the patients and their treating physician.

All patients gave their written consent to discuss their case in the MTB setting. According to the Bavarian Hospital Act (BayKrG) in the version of 28 March 2007 (GVBl. p. 288, BayRS 2126-8-G Art. 27 Para. 4), an ethics declaration for the anonymized retrospective evaluation of the data with the aim of quality assurance is not required.

*Input data preparation (“prompting”)*

The information provided to clinicians, on which the MTB structured treatment recommendations were based, was converted into a standardized prompt **(Supplemental Table 2)**. The input data for ChatGPT 4.0 were provided on the premise of educational case discussions. ChatGPT was assured that clinical treatment decisions were not based on its recommendations or research findings. All prompts were entered in English. The prompts were structured and designed to be as concise as possible.

*Analytical strategy:*

For analysis of ChatGPT’s performance a multilayer analytical strategy was developed. **Figure 1** gives an overview of the analysis pipeline. ChatGPT 4.0 was used for all analyses. Firstly, the established and guideline-based recommendations for therapy were analyzed as control in three common cancer entities (standard cases). ChatGPT was prompted to output first to last line therapy recommendations and evaluated for correctness. The complete output transcript is found in **Supplemental Table 3**. Correct treatment options were based on NCCN guidelines from 2021: 17, 18, 19

1. Left-sided colorectal cancer (CRC), UICC stage IV, microsatellite stable (MSS), *BRAF* wildtype (WT), *RAS* mutated (mut) (p.G12A)
2. Non-small cell lung cancer (NSCLC), UICC stage IV, programmed cell death ligand-1 (PD-L1): TPS 15% (TC 0), CPS 15%, ICS < 1 (IC 0), Exon 19 deletion (del): *EGFR* p.E746_A750del
3. Breast Cancer (BC), (invasive-lobular breast cancer), UICC stage IV, human epidermal growth factor receptor 2 (HER2/neu) negative, estrogen receptor (ER) 12/12, progesteron receptor (PR) 12/12, MSS

Chat GPT 4.0’s recommendations were analyzed in triplicate, with each replicate reflecting independent runs of the model for a respective standard- and MTB-case presentation to evaluate for congruency and potential adaptation of answers (**Figure 1**).

Cases from the MTB were also evaluated in triplicates accordingly. Both for standard and the MTB cases, generated responses were reviewed for accurate reproduction of provided information and disease specifications. Deviations were recorded as breach of concordance (BOC), equivalent to a failure to understand the question. In case of a BOC, the error was pointed out to ChatGPT, and the question was repeated with the identical prompt. In case of a second BOC, the question was rephrased until accurate reproduction was reached. BOC was recorded with numerical labeling in dependence of the number of failures including the first repetition. The full transcript of answers for the MTB cases is found in **Supplemental Table 4**.

The responses generated were prompted to be outputted as structured data, i.e. as a data table with a column for the type of recommendation (diagnostic or therapeutic), a column for the rationale and source of recommendation given and the treatment lines listed as rows. The feedback function was disabled to ensure no adjustment of responses was made by automatic or manual review. Correctness of answers for standard cases was evaluated by comparing the output to the NCCN-approved guidelines. Based on predefined performance indicators, for MTB cases, the recommendations were assessed by two independent human expert reviewers. Both human expert reviewers were board-certified physicians in hematology / oncology and have a background in personalized oncology with an experience of at least three years.

*Performance metrics and statistical analysis*

Five key performance metrics were defined for evaluating ChatGPT’s recommendations for the MTB cases. Information density and quality were evaluated by the two independent experts:

1. Recommendation type: diagnostic and therapeutic recommendations were counted on a per case basis both for the human MTB (hMTB) and the ChatGPT triplicates.
2. Information density: An information density index (IDM) was defined to allow for an evaluation of depth of information. The IDM was defined as the number of correct therapy recommendations divided by the number of correct and incorrect recommendations + 1 with resulting values ranging between 0 and 0.99:
3. Information consistency and variability of triplicates was addressed by measuring Fleiss’ Kappa for IDM scores20. Interpretation of Fleiss’ Kappa is similar to Cohen’s Kappa (**Supplemental Table 5).** Fleiss' Kappa is a statistical measure that quantifies the level of agreement among multiple raters when assigning categorical ratings to a set of items or subjects, while accounting for the agreement that would be expected by chance alone (Fleiss-Kappa = 0). Repetitive answers were only considered once. Synonymous answers are defined in **Supplemental Table 5**.
4. Information quality: The quality of recommendations given was captured via the analysis of the LoEs: LoEs were only assigned to recommendations that received a positive IDM score, i.e. were evaluated as correct therapy option. LoE categories were ordinally scaled with the highest evidence level (1A) assigned a score of 7 and the lowest (4A) assigned a score of 1. Statistical analyses included calculating the mean and standard deviation of LoE score differences between ChatGPT and hMTB for each case across the replicates. Average differences of values between ChatGPT and hMTB were calculated. For the visual representation, horizontal bar plots were generated. This approach provided a clear depiction of the alignment or divergence of ChatGPT recommendations compared to the hMTB. Cases with missing data were labeled as "data incompl." to indicate incomplete data for at least two replicates, thereby highlighting variability in the model's performance and data availability. Cases with incomplete data for both reviewers in the hMTB group were excluded, i.e. cases where no LoE was assigned.
5. Process efficiency: In the MTB context, we defined efficiency as the ability to achieve the maximum output with the minimum input of time and physicians allocated to work on a case. The time required to generate treatment recommendations was assessed on a case-by-case basis by comparing the time required to generate treatment recommendations using ChatGPT and the time required by individual investigators. The time spent by the hMTB was defined as the time from reviewing the information, including annotation and literature research, to writing the structured MTB recommendation. The mean of the time taken by the two human experts was used for comparison. The time required for the ChatGPT-derived recommendations included the time from receiving the information, formulating the prompt, refining prompts repeatedly if necessary, and reviewing the output. The time required for the cases was compared using a non-parametric Mann-Whitney U test.

**Supplemental Table 1**

Levels of evidence according to Horak, P. *et al.*

**Supplemental Table 2**

Overview of Prompts

**Supplemental Table 3**

Prompts and Answers for Standard Cases

**Supplemental Table 4**

Full transcript of answers

**Supplemental Table 5**

Interpretation categories and synonym recommendations for Fleiss’ Kappa agreement analysis in treatment decision studies
