# Supplementary material for: Large language model processing capabilities of ChatGPT 4.0 to generate molecular tumor board recommendations—a critical evaluation on real world data
Source: Oncologist. 2025 Sep 18;30(10):oyaf293. doi: 10.1093/oncolo/oyaf293 (PMC12557318; doi:10.1093/oncolo/oyaf293)
Supplement: oyaf293_Supplementary_Data [file oyaf293_supplementary_data.zip › Supplemental_Table 5.pdf]

Supplemental Table 5

Patient characteristics of the molecular tumor board cohort

Variable	Patients, No. (%) (N = 20)
**Age, median (IQR) [range]**	56 (42-76) [32-78]
**Sex**	
Female	10 (50%)
Male	10 (50%)
**Entities**	
Breast	3 (15%)
Bowel	3 (15%)
CNS/Brain	3 (15%)
Biliary	2 (10%)
Cervix	1 (5%)
Ovary	1 (5%)
Carcinoma of unkown primary (CUP)	1 (5%)
Esophagus	1 (5%)
Bone	1 (5%)
Head and Neck	1 (5%)
Prostate	1 (5%)
Lung	1 (5%)
Skin	1 (5%)
**Type of Sequencing Panel**	
Ampliseq Focus Panel	17 (80%)
Ampliseq BRCA Panel	3 (14%)
POLE-Mutational Analysis	1 (5%)
**No. of variants, median (IQR) [range]**	2 (1-2) [1-9]
**Total number of variants**	39
