# Supplementary material for: Large language model processing capabilities of ChatGPT 4.0 to generate molecular tumor board recommendations—a critical evaluation on real world data
Source: Oncologist. 2025 Sep 18;30(10):oyaf293. doi: 10.1093/oncolo/oyaf293 (PMC12557318; doi:10.1093/oncolo/oyaf293)
Supplement: oyaf293_Supplementary_Data [file oyaf293_supplementary_data.zip › Supplemental_Table 6.pdf]

Supplemental Table 6

Characteristic	MTB-cohort UKA [no.]	Höfflin et al. 2021 [no.] *	GENIE v18.0-public (2025) [no.]
Total	20 (100%)	488 (100%)	250.018 (100%)
Sex	Female 10 (50%), Male 10 (50%)	Female 230 (47.1%), Male 258 (52.9%)	Female 110105 (52.1%), Male 94633 (44.7%)
Median age [range]	56 [32–78]	54 [1–88]	61 [<1 – >89] **
Lower GI tract	3 (15.0%)	68 (13.9%)	21844 (8.7%)
Pancreas	0 (0.0%)	50 (10.2%)	10615 (4.2%)
Upper GI tract	1 (5.0%)	42 (8.6%)	6864 (2.7%)
Central nervous system	3 (15.0%)	45 (9.2%)	1759 (0.7%)
Unknown Primary Site	1 (5.0%)	37 (7.6%)	7945 (3.2%)
Hepatobiliary	2 (10.0%)	30 (6.1%)	4977 (2.0%)
Thyroid	0 (0.0%)	30 (6.1%)	3378 (1.4%)
Soft tissue and bone ***	1 (5.0%)	37 (7.6%)	1606 (0.6%)
Gyn (others)	1 (5.0%)	18 (3.7%)	1206 (0.5%)
Head and neck	1 (5.0%)	19 (3.9%)	2832 (1.1%)
Breast	3 (15.0%)	21 (4.3%)	20768 (8.3%)
Urogenital	1 (5.0%)	12 (2.5%)	8438 (3.4%)
Ovary	1 (5.0%)	12 (2.5%)	8916 (3.6%)
Dermatologic	1 (5.0%)	18 (3.7%)	9393 (3.8%)
Hematologic	0 (0.0%)	17 (3.5%)	n/a
Lung	1 (5.0%)	16 (3.3%)	36069 (14.4%)
Neuroendocrine	0 (0.0%)	10 (2.0%)	n/a
Other	0 (0.0%)	6 (1.2%)	n/a

* Tumor-type category names from Höfflin et al. were harmonized to match AACR Project GENIE category labels for comparability minor differences in grouping may therefore occur.
** median age for the GENIE cohort is taken from Pugh et al., 2022 as demographic was not recalculated for v18.0.
*** 'Soft tissue and bone' in Höfflin includes multiple sarcoma subtypes; the MTB-UKA cohort included only osteosarcoma.
