# Supplementary material for: Large language model processing capabilities of ChatGPT 4.0 to generate molecular tumor board recommendations—a critical evaluation on real world data
Source: Oncologist. 2025 Sep 18;30(10):oyaf293. doi: 10.1093/oncolo/oyaf293 (PMC12557318; doi:10.1093/oncolo/oyaf293)
Supplement: oyaf293_Supplementary_Data [file oyaf293_supplementary_data.zip › Supplemental_Table 7.docx]

**Supplemental Table 7**

## ESCAT-Stratified Actionable Mutations

| **Tumor / Case** | **Mutation** | **ESCAT Tier** |
| --- | --- | --- |
| **Case MTB 1** | | |
| **Breast** | PIK3CA H1047L | IA |
| **Case MTB 2** | | |
| **Cervix** | ALK R672S | IIIA |
|  | MAP2K1 F53L | IV |
| **Case MTB 3** | | |
| **Bowel** | PIK3CA H1047R | IIIA |
|  | KRAS G12D | IV |
| **Case MTB 4** | | |
| **Breast** | MYC amplification (copy number 5) | V |
| **Case MTB 5** | | |
| **Breast** | BRCA2 K1691AfsTer15 | IA |
|  | ESR1 D538G | IIA |
| **Case MTB 6** | | |
| **Biliary** | IDH2 R172G | IA |
|  | MET Y71C | IV |
| **Case MTB 7** | | |
| **Ovary** | PIK3CA C420R | IIB |
|  | BRCA2 K3326* | IIIB |
|  | ERBB2 R633Q | IV |
| **Case MTB 8** | | |
| **Biliary** | IDH1 R132C | IA |
| **Case MTB 9** | | |
| **CNS/Brain** | IDH1 R132H | IA |
|  | PIK3CA C420R | IV |
| **Case MTB 10** | | |
| **CNS/Brain** | PIK3CA P104L | IV |
| **Case MTB 11** | | |
| **CUP** | PIK3CA E365K | IIIB |
|  | MYC N26S | V |
| **Case MTB 12** | | |
| **Bowel** | PIK3CA E545K | IIIA |
|  | KRAS Q61H | IV |
| **Case MTB 13** | | |
| **Esophagus** | BRCA2 L1908R | IB |
|  | ATM P292L | IIIB |
|  | CCND1 amplification | IV |
|  | FGF19 amplification | IV |
|  | FGF3 amplification | IV |
|  | FGF4 amplification | IV |
|  | ASXL1 R676E | V |
|  | FAT1 Y3949 | V |
|  | TP53 | V |
| **Case MTB 14** | | |
| **Bone** | PIK3CA H1047R | IV |
| **Case MTB 15** | | |
| **CNS/Brain** | CTNNB1 amplification | IV |
|  | PIK3CA amplification | IV |
|  | RAF1 amplification | IV |
|  | DCUN1D1 amplification | V |
| **Case MTB 16** | | |
| **Head and Neck** | BRAF I592N | IV |
| **Case MTB 17** | | |
| **Prostate** | BRAF K601E | IIIB |
| **Case MTB 18** | | |
| **Bowel** | POLE P286R | IIB |
|  | PIK3CA R1023Q | IIIA |
|  | PIK3CA R88Q | IIIA |
|  | EGFR E758K | IIIB |
|  | ALK F1193L | IV |
|  | JAK1 T688P | IV |
|  | RET I913S | IV |
|  | ROS1 E1958Ter | IV |
|  | DCUN1D1 R13C | V |
| **Case MTB 19** | | |
| **Lung** | ERBB2 Y772_A775dup | IA |
| **Case MTB 20** | | |
| **Skin** | NRAS Q61R | IB |

ESCAT-stratified actionable alterations for all 20 cases. For each case, the tumor type, actionable genomic alteration(s), and assigned ESCAT tier(s) are listed. ESCAT tiers follow the original ESMO framework [Mateo et al., 2018] and reflect the most recent evidence available as of 2024, incorporating tumor-specific clinical relevance. Cases are grouped by molecular tumor board (MTB) number, with multiple alterations per case shown where applicable.
