# Supplementary material for: Large language model processing capabilities of ChatGPT 4.0 to generate molecular tumor board recommendations—a critical evaluation on real world data
Source: Oncologist. 2025 Sep 18;30(10):oyaf293. doi: 10.1093/oncolo/oyaf293 (PMC12557318; doi:10.1093/oncolo/oyaf293)
Supplement: oyaf293_Supplementary_Data [file oyaf293_supplementary_data.zip › Supplemental_Table 8.pdf]

Supplemental Table 8

Gene	MTB-cohort UKA [no.]	Höfflin Panel [no.]	GENIE v18.0-public [no.]
PIK3CA	10 (50%)	63 (28.5%)	29814 (10.1%)
BRCA2	3 (15%)	7 (3.2%)	14163 (4.5%)
ALK	2 (10%)	5 (2.3%)	9012 (2.9%)
BRAF	2 (10%)	19 (8.6%)	14392 (5.3%)
ERBB2	2 (10%)	21 (9.5%)	9328 (2.8%)
IDH1	2 (10%)	8 (3.6%)	6612 (2.5%)
KRAS	2 (10%)	32 (14.5%)	35153 (13.6%)
MGMT	2 (10%)	0 (0%)	322 (1.4%)
ASXL1	1 (5%)	0 (0%)	9922 (3.9%)
ATM	1 (5%)	86 (38.9%)	19350 (5.7%)
DCUN1D1	1 (5%)	0 (0%)	298 (0.3%)
EGFR	1 (5%)	52 (23.5%)	16811 (5.4%)
ESR1	1 (5%)	0 (0%)	4648 (1.9%)
FAT1	1 (5%)	0 (0%)	18854 (6.1%)
IDH2	1 (5%)	2 (0.9%)	2879 (1.2%)
JAK1	1 (5%)	0 (0%)	4973 (1.8%)
NRAS	1 (5%)	19 (8.6%)	7436 (2.8%)
MAP2K1	1 (5%)	0 (0%)	2232 (0.9%)
MYC	1 (5%)	0 (0%)	2586 (0.9%)
POLE	1 (5%)	0 (0%)	9473 (3.7%)
RET	1 (5%)	33 (14.9%)	6529 (2.2%)
ROS1	1 (5%)	0 (0%)	11598 (3.6%)
TP53	1 (5%)	87 (39.4%)	106590 (35.0%)
MET	1 (5%)	36 (16.3%)	7393 (2.3%)
APC	0 (0%)	128 (57.9%)	32354 (8.9%)
ERBB4	0 (0%)	72 (32.6%)	10729 (3.2%)
SMAD4	0 (0%)	79 (35.7%)	10241 (3.6%)
