# Supplementary material for: Large language model processing capabilities of ChatGPT 4.0 to generate molecular tumor board recommendations—a critical evaluation on real world data
Source: Oncologist. 2025 Sep 18;30(10):oyaf293. doi: 10.1093/oncolo/oyaf293 (PMC12557318; doi:10.1093/oncolo/oyaf293)
Supplement: oyaf293_Supplementary_Data [file oyaf293_supplementary_data.zip › Supplemental_Table 10.docx]

**Supplemental Table 10**

ESCAT Tier Definitions (adapted from Mateo et al., 2018)

- Tier I: Alterations with ready-for-practice clinical utility.
  - IA: Target–drug pairs with proven benefit in randomized clinical trials and regulatory approval for the same tumor type.
  - IB: Target–drug pairs with proven benefit in randomized trials but approved for a different tumor type (tumor-agnostic approval or strong cross-tumor evidence).
- Tier II: Alterations with probable clinical utility.
  - IIA: Clinical benefit shown in prospective trials, but not yet confirmed in randomized phase III trials.
  - IIB: Clinical benefit supported by strong retrospective or early-phase prospective data.
- Tier III: Alterations with investigational utility.
  - IIIA: Clinical activity shown in small studies without definitive evidence of patient benefit.
  - IIIB: Preclinical or biological rationale with preliminary clinical evidence.
- Tier IV: Preclinical evidence of actionability with no supporting clinical data.
- Tier V: Alterations for which there is evidence of lack of clinical utility.
