# Supplementary material for: Large language model processing capabilities of ChatGPT 4.0 to generate molecular tumor board recommendations—a critical evaluation on real world data
Source: Oncologist. 2025 Sep 18;30(10):oyaf293. doi: 10.1093/oncolo/oyaf293 (PMC12557318; doi:10.1093/oncolo/oyaf293)
Supplement: oyaf293_Supplementary_Data [file oyaf293_supplementary_data.zip › Supplemental_Table_11.pdf]

Supplemental Table 11

Definitions of Qualitative Variability Categories

Label	Definition
novel drug class introduced	addition of drug class, present in one output but absent in others (addition of new class)
omission of previously recommended drug class	a drug class present in at least two outputs but omitted in another (forgotten or lost drug class)
evidence overreach	suggestion with weak clinical support or off-grid rationale (e.g., ALK inhibitors for novel ALK mutations, BRAF inhibitors for undescribed BRAF variants)
hallucination	confidently stated recommendation that is factually incorrect, unsupported by evidence, or contextually inappropriate—especially when it appears plausible
diagnostics expansion	output includes expanded or advanced diagnostics not suggested in all outputs (e.g., NGS, rebiopsy)
trial enrollment	output includes an explicit recommendation for clinical trial participation, not repeated in other replications
best supportive care	suggestion of best supportive care as a management strategy, not present in all replications
